# Do robots outperform humans in human-centered domains?

**DOI:** 10.3389/frobt.2023.1223946

**Published:** 2023-11-07

**Authors:** Robert Riener, Luca Rabezzana, Yves Zimmermann

**Affiliations:** ^1^ Sensory-Motor Systems Laboratory, Institute of Robotics and Intelligent Systems, ETH Zurich, Zurich, Switzerland; ^2^ Paraplegic Center, University Hospital Balgrist, University of Zurich, Zurich, Switzerland; ^3^ Robotic-Systems Laboratory, Institute of Robotics and Intelligent Systems, ETH Zurich, Zurich, Switzerland

**Keywords:** humanoids, robotics, human performance, human robot comparison, walking robots, service robotics, robotic structures, robotic functions

## Abstract

The incessant progress of robotic technology and rationalization of human manpower induces high expectations in society, but also resentment and even fear. In this paper, we present a quantitative normalized comparison of performance, to shine a light onto the pressing question, “How close is the current state of humanoid robotics to outperforming humans in their typical functions (e.g., locomotion, manipulation), and their underlying structures (e.g., actuators/muscles) in human-centered domains?” This is the most comprehensive comparison of the literature so far. Most state-of-the-art robotic structures required for visual, tactile, or vestibular perception outperform human structures at the cost of slightly higher mass and volume. Electromagnetic and fluidic actuation outperform human muscles w.r.t. speed, endurance, force density, and power density, excluding components for energy storage and conversion. Artificial joints and links can compete with the human skeleton. In contrast, the comparison of locomotion functions shows that robots are trailing behind in energy efficiency, operational time, and transportation costs. Robots are capable of obstacle negotiation, object manipulation, swimming, playing soccer, or vehicle operation. Despite the impressive advances of humanoid robots in the last two decades, current robots are not yet reaching the dexterity and versatility to cope with more complex manipulation and locomotion tasks (e.g., in confined spaces). We conclude that state-of-the-art humanoid robotics is far from matching the dexterity and versatility of human beings. Despite the outperforming technical structures, robot functions are inferior to human ones, even with tethered robots that could place heavy auxiliary components off-board. The persistent advances in robotics let us anticipate the diminishing of the gap.

## Introduction

### Motivation

During the last decades, society has been confronted with an incessant advance of robotictechnologies in the human work environment. First, the production industry deployed robots for well defined, heavy lifting, and high accuracy assembly tasks that are characterized by a high number of repetitions, e.g., in the automotive industry. Robots with more versatile functions were not existing or reliably functioning. Now, robots advance more and more into the field of service tasks that require more demanding behaviors with respect to perception, adaptation, and cooperation with humans, in human-centered domains such as private households or clinical facilities. Latest developments in humanoid and legged robotics demonstrate that technology advances to accomplish an increasing number of functions that are typical for humans (Miki et al., 2022; Boston Dynamics Support Center, 2021; Boston Dynamics, 2017). Furthermore, parathletes such as Heinrich Popow or Markus Rehm are using high-tech lower leg prostheses for sprint and broad jump disciplines, respectively. Their performances belong to the best among all athletic achievements, including those without amputations (Hassani et al., 2015; Beckman et al., 2017).

Humanoids and legged robots in general experienced impressive advancement in the last decades. The story of legged robots started, e.g., with Mark Raibert’s hopping machines in the 1980ies (Raibert, 1986) or Honda’s Asimo in the 1990ies (Sakagami and Watanabe). Since these first endeavors in the field, remarkable improvements were made afterwards. Very powerful und skilled walking and running robots have been introduced in the last decade with robots such as Atlas, MIT Cheetah, ANYmal, Spot, or Cassie (Boston Dynamics, 2022; Seok et al., 2014; Hutter et al., 2016a; Boston Dynamics Support Center, 2021; Gong et al., 2019) and its commercially available successor Digit (C et al., 2021; NED Directory, 2023; Robots, 2019). Performance of robots showcased at events such as the DARPA Robotics Challenge 2015 and 2021 (Johnson et al., 2015), the ANA Avatar XPRize (Luo et al., 2022) or the Cybathlon 2016 and 2020 (Riener, 2016; Wolf and Riener, 2018; CYBATHLON, 2022); they show impressive technological achievements close to being applicable in daily live.

This impressive advancements in robotics together with the progressive rationalization and outperformance of human labor by robotic technology induces resentment or even fear in society (Nestik et al., 2018), whereas stirring up hope to cede all dangerous and wearing tasks to machines. The image of robots as superior to humans has also been pushed by the entertainment industry with movies such as Terminator, Ironman, or I-Robot. This might foster high expectations for robotic technology, thereby creating the public belief, that humanoid robots and artificial limbs are readily available, and that they can fully replace or even outperform the functions of our biological limbs.

So where are we today? Do robots already outperform human beings with respect to sensory, computational, and motor performance? Can robots now replace humans acting in typical human-centered domains? This paper attempts to shine a light into this pressing question by comparing state-of-the-art robotic performance with that of human beings.

### Goal

The goal of this study is to perform an objective (i.e., a normalized) comparison between existing humanoid robotic devices with human beings with respect to their structures and functions in human domains. Structures include sensory, computational, and motor components in both robots and humans. They are required to enable functions such as locomotion and haptic interactions within these domains.

Comparisons of robotic systems with biological ones is not a straight-forward process, because the technical systems are not built by biological cells that can regenerate, grow, and adapt to (slowly) changing conditions. On the other hand, technology can easily outperform a human when focused on a single and repetitive function, as the technology can be optimized or just enhanced with respect to performance of only a single function. For example, a robot can become stronger and faster, when choosing larger and more powerful actuators. A robotic arm can also reach higher when choosing longer links. And, a robot can work longer and thus highly repetitive, without fatigue and endurance limitations like in humans, when choosing larger batteries with higher capacities or when harvesting external energy sources, e.g., solar energy. However, all these enhancements come at a cost: the robotic system usually becomes much larger or heavier (or both) than a human, which limits other functions such as agility, portability, dexterity, or versatility.

Therefore, it is important when conducting any type of functional or structural comparison to relate a concrete, measurable feature (i.e., a physical quantity), against a respective normative base, a common “denominator,” such as size, mass, available (battery) power, or cost of transport (Tucker, 1975; Kashiri et al., 2018). With such a normalization, the relation of the feature to the denominator is approximated to be linear, which should be an accurate estimation in close proximity to the normalization point and is commonly performed to compare features at different scales, e.g., power to weight ratio. The comparison refers to environments and tasks that are made and/or accessible for human beings and serve them in their everyday leisure or work lives.

Furthermore, robot and human performance must be compared in similar environments, while doing similar tasks. For example, the performance of a vacuum cleaning task should not be assessed with measures relevant for outdoor locomotion or performance in the assembly line of a car manufacturer. We focus this review work on humanoid robots, to allow a “fair” (apples to apples) comparison and to limit the scope of the comparison. We assume that general structures, comprising legs and limbs in a slender appearance, can act within human-centered domains and interact with humans for a series of different human-like tasks.

Related work mostly focused on comparisons of specific structural components between technological and biological systems. Aubin et al. (2022) investigated embodied energy storage. Rothemund et al. (2021) discuss the benefits of flexible structures in biological systems and the potential of material science to provide technological counterparts. Yin et al. (2021) review control and perception methods for deformable object manipulation. Furthermore, many publications compare the performance of biological and technological actuation systems (Higueras-Ruiz et al., 2021; Liang et al., 2020; Acome et al., 2018). Skorka and Joseph (2011) compared visual sensors. Yamane and Murai (2016) discussed the similarities and differences between human and humanoid robot structures qualitatively. A comprehensive comparison of structures and functions between the human and humanoid robots is not known to the authors.

## Method of comparison

### Comparing apples with apples

It is neither trivial nor meaningful to compare, for instance, the speed of an autonomous robotic car with that of a sprinting human; the costs of machine maintenance with the costs of sustaining human life; or aspects of technical deterioration with biological aging. Moreover, a robot devoid of feelings cannot be compared to the human experience of emotion. Robots, however, can move, perceive, and communicate, but at present they are incapable of experiencing an emotional spectrum, for example, feelings of happiness after successfully completing a task or frustration in times of failure. Since robots do not yet have an emotional framework, comparison in this context is not meaningful as the tasks and contexts differ.

**FIGURE 1:**
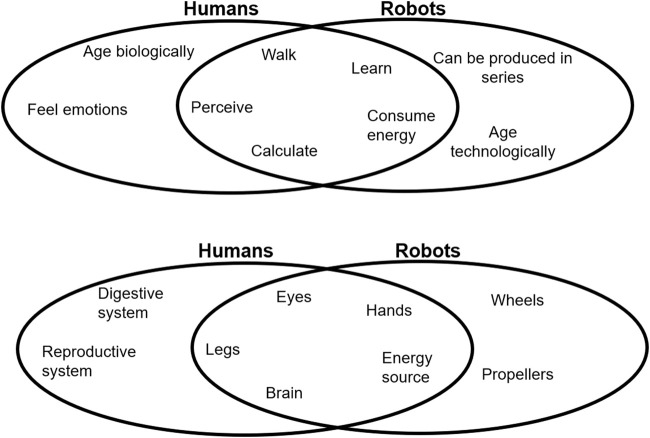
Overlap of functions and structures in human and robotic domains.

Robots are technological systems developed with the purpose of serving humans, and in some cases, they do this by replacing human operators. Therefore, we compare “apples with apples”, i.e., similar “structures” that perform “functions” of similar activities (Figure 1) and take place in similar “domains”, shared by humans and robots. Such meaningful comparisons are those of walking or running (on legs) in human-centered domains, grasping, manipulating objects relevant for daily life, learning new movements, perceiving the environment, and processing this information with some level of “cognition,” computing a motor task, or consuming energy for comparable motor tasks to name just a few.

Comparing robots and humans with respect to their common structures and functions in similar tasks and contexts allows us to refer to applications, where a robot can support or replace humans or a part of the human body to serve society in any task that is relevant for aspects of our private or work lives. Therefore, we focus on “service functions” for humans and include movement and interaction tasks in human-centered domains, elaboration, and communication of information perceived from such domains. Our human domains have evolved in a way that we can function well within these domains with our structures including legs, arms, fingers, eyes, ears, etc. Therefore, we assume that robotic structures that serve to support humans in their domains must have humanoid structures, and consequently, we focus on robots with legs and hands, with all the sensors carried by the robot. Structures and functions that are required for self-sustaining, such as breathing, cardiovascular functions, eating, or digestion, are not taken into consideration (Figure 1).

In this paper, we first confront anatomical structures such as bones, joints, muscles, eyes, brain etc. with corresponding technical systems like links, bearings, actuators, cameras, and processing units by comparing features such as Young Modulus, friction, force, power generation, power consumption or spatial and temporal resolution. Then we move toward humanoid functions that are based on the presented structures. For example, given the common function “locomotion,” we compared features such as walking or running speed.

### Human-centered domains

The performance of humans and robots regarding their functions and structures must be compared with respect to defined domains and related tasks. The domains and tasks must allow a replacement of humans or parts of humans by robots. Examples of domains are indoor environments or rooms, outdoor environments, environments that involve objects, devices, and machines (e.g., cars to drive, buses to enter, buttons to press, or signs to read) or other humans with whom robots would need to interact (e.g., to listen, to talk, or to communicate).

We can distinguish natural and artificial conditions of domains. Typical natural environmental conditions to be considered are exposure time for service and resting, environmental temperature, inclination of the ground, nature of the ground (such as texture, gravel, or sand), friction between feet and natural ground (dry, wet, snow, or ice), exposure to radiation, water or any other media, brightness, etc. Artificial environmental conditions are room temperature, type and intensity of room lighting, and obstacles to be negotiated (e.g., slippery artificial floors, steps, escalators, or doors). Further, conditions are determined by the properties of objects and devices that require interaction.

In this paper, we target a generic comparison between robots and humans in natural and artificial domains that confront us in daily life conditions. Thus, the natural environmental conditions chosen are 24 h service and rest cycles with 8–16 h of service exposure, and temperature that can vary between −10°C and 40°C depending on location, season, and time of the day. We compare walking grounds to include flat and rough terrains, dry, sticky, or even slippery due to snow or ice. Brightness resulting from the typical outdoor light conditions during daytime, 100′000 lux on average (Kelley et al., 2006), and 0.0001 lux at nighttime (Engineering Toolbox, 2022a).

Artificial conditions considered in this study are typical indoor environments that comprise even terrains on flat floors but may also contain steps or stairs, barriers such as doors with handles, and objects such as chairs or tables. The human or robot can interact with different objects and interfaces of vehicles that are actively driven by the user, or that passively transport the user to different locations. Room temperature is in the range of about 18°C–32°C (World Health Organisation, 2018).

Our comparisons focus on robots and humans analyzed in usual conditions of industrialized societies. Therefore, we assume augmentative technologies such as shoes, clothes, and eyeglasses, that can enhance human performances in special indoor and outdoor environments, as “natural” parts that belong to the humans. In contrast, powered devices worn or carried by humans to enhance their performance and often include some level of technical intelligence, such as smartphones, exoskeletons, bikes, and other means of transportation are excluded in this study.

### Functions and structures are derived from ICF standard

The International Classification of Functioning, Disability and Health (ICF) released by the WHO (World Health Organisation, 2007) can be used as a starting point for collecting different functions and structures of both humans and robots. The ICF distinguishes between human body structures (joints, limbs, or organs, etc.), body functions (joint movement, leg movement, vision, etc.), activities related to tasks and actions of an individual (walking, using public transportation, or cooking) and participation as an involvement in a life situation (visit lectures, participate and cultural events, or playing in a music band). These aspects are viewed as a complex interaction between the personal factors of the individual and the contextual factors of the domain.

The starting point for the choice of the compared functions and structures is a human being as classified by the ICF. Starting with the human helps us finding the concrete robotic functions and structures out of a virtually infinitive number of different robotic designs.

The ICF itself highlights the importance of environmental factors and lists the most relevant in the classification of human structures and functions.

To compare the functions and structures of robots and humans quantitatively, measurable “features” have been introduced–whenever possible. Examples of features to evaluate functions are gait speed for walking, typing speed - rate for writing on a keyboard, or words per minute for speaking. Examples of features to evaluate structures are mass for a body segment or the entire body, image resolution for the vision system, or power density (e.g., power/mass ratio) for an actuator.

Functions to be compared are derived from ICF functions and include aspects of activities and participation (World Health Organisation, 2007) and applied to the environment and the robot representatives. In this study, we collected robots and their applications with respect to the following functions:

• Movement: Walking, running, climbing stairs, changing, and maintaining basic body positions, negotiating rough terrain, crawling, jumping, and swimming.
• Physical interaction: Driving a vehicle, lifting, and carrying objects with upper extremities, kicking objects with the legs, and use of hands and arms.
• Elaboration: Basic motor learning

In addition, the non-haptic interaction functions are presented in Supplementary Appendix SB:

• Elaboration: attention and multi-tasking functions, reading, and writing.
• Communication: Conversation, i.e., listening and producing verbal and non-verbal messages

These functions are resulting out of a complex interplay of sensory, motor and cognitive components and their sub-functions–in both humans and robots. The execution of functions depends on the existence of physical structures. Structures were derived from the above-listed functions performed by the human or robot, as defined by the ICF, and include:

• Sensory structures: for vision (eye and cameras), tactile perception (skin and tactile sensors), proprioception, and balance (vestibular structures of the inner ear and inertial measurement units)
• Motor structures: for actuation (muscles and actuators)

In addition, the structures for non-haptic interaction and computational abilities are presented in Supplementary Appendix SB.

• Motor structures: speaking (organ of speech and speakers)
• Sensory structures: hearing (ears and microphones)
• Computational structures: for memory and computation (brain and CPU/storage devices.

### Denominators

We normalize the features by common denominators and make them comparable among different systems. For example, the payload of a large and heavy-lifting robot used for construction should be downscaled (i.e., normalized) by using a denominator that takes care of the size of the system. Like features, denominators should be measurable, so that they are applicable for normalization. Denominators can be picked among those features of functions and structures that are usually not the primary focus for the design of the robot or the evolution of the human. They are rather a consequence of the physical implementation of the primary design features that are required to obtain desired (e.g., service) functions. Measurable features of functions and structures were compared (i.e., normalized) with respect to the following denominators:

• Mass, in *kg.*
• Size, in *m*, for example, body height, main dimension, nominal length.
• Power consumption, usually in *W*, including dissipated power.
• The inverse of the operational time (under normal conditions), in *s* ^-1^. The inverse has been taken to get the same polarity of the respective feature to better compare the features in relation to different denominators.
• Cost of Transport (*CoT*), a dimensionless metric
• Material density, in *kg m* ^-3^.

Cost of transport *CoT* (or specific resistance) is a common normalized metric that refers to energy consumption for locomotion (Kashiri et al., 2018; Tucker, 1975):

$$\mathrm{CoT}=\frac{E}{mgd}=\frac{P}{mgv},$$

where *E* is the energy inserted into the system, *m* the mass of the system, *d* the distance it travels, *g* the gravity acceleration, *v* the velocity, and *P* the input power.

The linear dashed line drawn in many of the figures, symbolizes a linear scaling of the feature (of certain functions or structures) with respect to a denominator based on the values found for the human. Values of robots that are placed above this dashed lines mean that the robot performs “better” than the human with respect to the single feature taken into account, with respect to the denominator chosen. The application of denominators to “normalize” the features in a linear way based on a linear scaling of the feature quality using the denominator. For many of the features the relation to the denominators is non-linear. In these cases, the comparison of human and robot features by the linear approximation is only reliable in close proximity to denominator values typical for humans. Thus, we indicate the 2^nd^ percentile female to 98th percentile male interval for each denominator as indication for the magnitude of scaling.

### Choice of robots and human representative

Out of a collection of thousands of different service robots in existence today, we have chosen those that fulfill the requirements to be in principle able to function in human-centered domains:

• Legged robots excel in negotiating rough terrain by leveraging their ability to select footholds and cross gaps (TheIHMC, 2019). Thereby, they can reach locations that similarly sized wheeled-only systems struggle with, while having less impact on the soil. Furthermore, the capability of stepping rather than rolling over obstacles is a significant advantage in narrow and cluttered home environments and environments that include steps and stairs. Therefore, we restrict the comparison to legged robots. This includes bipedal robots, such as Asimo (ASIMO, 2022) or Atlas (Boston Dynamics, 2022), four-legged robots, such as Spot (Boston Dynamics Support Center, 2021) or ANYmal (Hutter et al., 2016a), and even legged robots with wheels, such as Handle (Boston Dynamics, 2017; IEEE Spectrum Robots, 2021) or Ascento (Ascento, 2022). Robots with wheels or tracks only are excluded, e.g., Eve [by Halodi Robotics AS, (Halodi Robotics, 2021)] or autonomous wheeled vehicles.
• The robots must be of slender appearance to fit through doors or move in narrow spaces, e.g., in human crowds. Very broad and bulky robots are excluded, such as BigDog [by Boston Dynamics, (Playter et al., 2006)].
• The robots must have sufficient body height to be able reach and grasp objects on tables and shelves. Assuming that an additional robotic arm can be added to reach higher, a minimum body height of 0.5 m might be sufficient. Smaller robots, such as Robotis OP 2 (Robotis, 2023) or JO-ZERO (ikinamo, 2009a), are excluded.
• The robots must be powered to perform energy demanding, daily tasks, such as opening a door and they must be strong enough to carry objects that are relevant for daily life activities. Therefore, weak or unpowered robots, such as passive walking machines (Collins et al., 2005) are excluded. Also flying drones are excluded due to the negative effects of the strong downwash.
• The robots must be able to perform simple manipulations. Therefore, they must be equipped with arms such as Atlas (Boston Dynamics, 2022), iCub (Beira et al., 2006), or Digit (C et al., 2021) or be able to get equipped with an arm or any manipulative device such as Cassie (Gong et al., 2019).
• The robots must have sensors to perceive the environment to perform locomotion and manipulation tasks robustly in dynamic environments.
• The robots must not produce too much noise or exhaust gases, as they would be not acceptable in human-centered indoor environments. Thus, machines such as BigDog are excluded (Playter et al., 2006).

In this study, we analyze 27 robots that match these criteria, such as iCub, Atlas, ASIMO, or Sarcos (see Table 1). Not all robots have human appearance, as they might have no trunk, shortened arms, small or no heads, etc. And the robots can comprise structures that are not existing in mammals such as shaft-hub connections or prismatic joints. A nice example of a (nameless) bipedal robot with prismatic knee joints has been presented by SCHAFT, Japan some years ago (Ackerman and Guizzo, 2016).

**TABLE 1:** Compared robots. List of robots considered in this study. The robots have been selected among those currently existing as of 2021. All are potentially capable of taking over human-centered service tasks. The numbers on the far right indicate the year of release of the respective robots.

Nr.	Name	Type	Notes	References	Year
1	ATRIAS	Biped	Walking robot only with torso, no arms. Inspired by a spring-mass model. ATRIAS = Assume the robot is a sphere	Hubicki et al. (2016); Dynamic Robotics Laboratory (2015); Dynamics Laboratories (2015)	2015
2	Asimo	Biped	Taken as reference for Honda robots. Superior to both E-Series and P-Series	ASIMO (2022)	2000
3	HR 18	Biped	Small sized humanoid prototype	Hemker et al. (2009)	2009
4	HRP	Biped	HRP-2 Model	Kaneko et al. (2011); Stasse et al. (2018)	2002
5	HUBO	Biped	HUBO 2	IEEE Spectrum (2010)	2009
6	iCub	Biped	Kid-sized humanoid	Beira et al. (2006), Romualdi et al. (2018), Romualdi et al. (2018), Pontes (2019), Fakultät für Elektrotechnik und Informationstechnik (2022), Tktronix (2022)	2009
7	LOLA	Biped	The most recent robot of the two tethered TUM bipeds Johnnie and LOLA.	IEEE Spectrum Robots (2022b)	2010
8	Mahru III	Biped	MAHRU III version, which can be connected to external AI.	Kwon et al. (2007)	2007
9	NAO	Biped	Doll-sized humanoid used in RoboCUP soccer competitions	Aldebaran Documentation (2020); Robots (2021); Aldebaran (2008); Cristiano et al. (2011)	2008
10	REEM	Biped	REEM humanoid model C	PAL Robotics (2022)	2013
11	Sarcos	Biped	Sarcos experimental humanoid (not the Sarcos exoskeleton)	Morimoto et al. (2007)	2008
12	Sony QRIO	Biped	Small sized humanoid prototype	IEEE Spectrum Robots (2018)	2003
13	Atlas	Biped	Last version of one of the best performing robots in the world	Boston Dynamics (2022); Smith Marshal and Marshal Smith Technology (2018)	2021
14	Toyota Partner	Biped	Humanoid running robot	Toyota Motor (2022); Ota (2010)	2000
15	WABIAN	Biped	Humanoid WABIAN model 2	Biped humanoid robot group (2013); Mu et al. (2012)	2006
16	Durus	Biped	Efficient gait robot with up to 8 h of battery life and 5 h of walking battery life	Wevolver (2022)	2013
17	Walk-Man	Biped	Built to operate in dangerous environments	Thomson Reuters (2015); Ackerman and Guizzo (2021); EurekAlert (2018); Instituto Italiano di Technologia (2018); Kashiri et al. (2018)	2015
18	Cassie	Biped	Maximum walking speed 2.1 m/s. For longer distance walk only 1.57 m/s achieved	Xie et al. (2019); Gong et al. (2019); IEEE Spectrum ROBOTS (2022a); Lundeberg (2021); Kashiri et al. (2018)	2016
19	TORO	Biped	10 kg payload. No running or fast walking. 250 W in steady state. Stair climbing only up to 5 cm steps	DLR (2022); Englsberger et al. (2014)	2013
20	Flame	Biped	Legs-and-torso-only walking robot	TU DELFT (2022); BotJunkie (2008); Hobbelen et al. (2008)	2005
21	Handle	Biped with wheels	With wheeled legs. It can reach high speeds and jump up to 1.2 m and carry 45 kg boxes. No information found, whether it can climb stairs	Boston Dynamics (2017), IEEE Spectrum Robots (2021)	2019
22	Ascento	Biped with wheels	With wheeled legs. It can step and climb stairs, and reach high rolling speeds; can jump up to 0.4 m. Battery life of 8 h	Ascento (2022); Zürich (2022)	2020
23	Spot	Quadruped	Quadrupedal robot. Maximum speed 1.6 m/s, able to run and trot	Boston Dynamics Support Center (2021); IEEE Spectrum Robots (2019)	2020
24	ANYmal	Quadruped	Quadrupedal robot for inspection and maintenance in industrial environments	Hutter et al. (2016a)	2016
25	Titan XIII	Quadruped	Sprawling type, all-terrain robot	Kitano et al. (2016)	2016
26	MIT Cheetah	Quadruped	One of the fastest running robot ever developed	MIT News | Massachusetts Institut e of Technology (2013)	2012
27	ANYmal on wheels	Quadruped with wheels	Quadrupedal robot for inspection, home delivery, and rescue missions	Bjelonic et al. (2020); Bjelonic et al. (2019); Swiss Mile (2022)	2020

To the best of our knowledge, none of these robots have been used in ordinary real-life scenarios perhaps with one exception: Just recently, robot Digit came out as the successor of Cassie (Gong et al., 2019). It is commercialized by the spin-off Agility Robotics. The company seems to lead the commercial development of humanoid robots with the aim of relieving humans from certain tasks and to compensate for the increasing decline in qualified workers as a result of demographic change. Most of the other robots are only available as lab devices that require extra efforts to be used outside the lab. Others do not comprise trunk and arms, which limits their functions. Even those robots that are commercially available, have not been tested in random real-life situations, but only in standardized “fake-like” settings, e.g., Atlas within the DARPA challenge. Others were used for entertainment shows and soccer challenges, e.g., Asimo or iCub. Tesla’s Optimus Robot (Youtube - Tesla Bot Update, 2023) is another promising humanoid released recently. However, not many technical specifications have been published yet, and despite the remarkable development efforts and results, experts were assessing the robot’s performance as not too spectacular (Ackerman and Guizzo, 2022). However, substantial improvements were introduced with the updated version (Youtube - Tesla Bot Update, 2023).

The human representative, the so called “reference man”, was defined according to the International Commission on Radiological Protection’s 1974, as someone being between 20 and 30 years of age, 170 cm of height, weighing 70 kg, and living in a climate with an average temperature of 10°C–20°C (Snyder et al., 1974). We have chosen this source because it provided a large amount of available data and is used and cited in many other projects. More recent models of the standard person confirmed similar values for average weight and height, with only slightly larger values for males (Ellis, 1990). An adult, average person consumes about 300 W–450 W for walking (Radhakrishnan, 1998; Das Gupta et al., 2019) and between 700 W and 1500 W for running depending on the speed (Riddick and Kuo, 2022). These values include the power consumption of both mechanical and metabolic activity. The parameter operational time is defined as the time an average adult can operate without needing a rest for recreation, sleep, or food intake. We estimate the operation time of an average human for walking to be about 8 h and for running to be about 1 h, which is a reasonable duration for a healthy adult who is jogging or engaging in moderate exercise.

## Comparison of structures

### Vision systems

The human eyeball has a diameter of 21–27 mm (Bekerman et al., 2014) and it weighs about 7.5 g (Todd et al., 1940). Power consumption due to retinal, metabolic, and muscular activities is less than 10 mW (Skorka and Joseph, 2011). The monocular visual field of a healthy adult extends maximally 135° in the vertical and maximally 160° in the horizontal directions (Skorka and Joseph, 2011). The spatial resolution of the eye is 576 MPX (Syawaludin et al., 2019) with highest resolution at the center and gradually declining resolution toward the periphery. The highest resolution is at least 1 arc minute (1/60)°, which corresponds to a visual acuity of 1 or 100%. The shortest duration required to detect unknown images is 13 m (Potter et al., 2014), which translates to a detection frequency of 77 Hz. Other publications estimate that in typical office conditions, the human eye can detect temporal changes at 65 Hz, and frequencies greater than 80–90 Hz at high luminance levels (Skorka and Joseph, 2011). The perceived light is in the visible spectrum characterized by wavelengths in the range of 380 nm–740 nm.

The human eye can adapt to different brightness conditions, yielding a very large dynamic range, or luminance, reaching from 10^–6^ cd/m^2^ in the dark (scotopic threshold) to 10^8^ cd/m^2^ during daylight (damage threshold) (Spillmann and Werner, 2012). Hence, over different scenes, a luminance range of 10^14^ can be detected by the human eye. Other sources state a luminance range of 10^10^ (Ferwerda, 2001). However, it requires several seconds to minutes until the human vision system adjusts to a new illumination condition. For a specific scene, the contrast ratio approaches 120 dB, which corresponds to a luminance ratio of 10^6^ (Rosenthal et al., 2004; Durini and Arutinov, 2020). Color vision, or photopic vision, occurs at luminance greater than 3 cd/m^2^. Vision at a luminance lower than 0.001 cd/m2, in which only rods are operative, is called dark vision, or scotopic vision–the corresponding luminance value is called “dark limit” (Skorka and Joseph, 2011).

Human depth perception is possible due to a variety of different cues, including static and dynamic monocular cues, oculomotor cues (accommodation and convergence), and binocular cues (stereopsis, i.e., binocular disparity) (Schiller et al., 2011; Riener and Harders, 2012). Based on the human ability to detect depth differences at 30 arcseconds disparity (Coutant and Westheimer), we estimate the resolution for depth perception at 10 m distance to be around 0.25 m using basic trigonometry.

Traditional RGB cameras based on charged-coupled device (CCD) or complementary metal-oxide-semiconductor (CMOS) chip technologies have a narrower dynamic range than the human eye, although the gap is closing. Skorka and Joseph (2011) have published a comparison between the human vision system and artificial vision sensors in 2011. At that time, some CCD image sensors achieved higher spatial resolutions and temporal resolutions than the human eye. On the other hand, dynamic ranges and dark limits were still significantly less than in the human eye with a difference of 1.6–4.5 orders of magnitude. Denominator values of power consumption, mass, and size were significantly higher than those of the human eye. Even when checking the data sheets of the newest CCD and CMOS cameras, these values have not improved.

The work from Skorka and Joseph did not include lens systems and sensors with extremely wide field of view (FOV) or very high framerate. “Fish-eye lenses” on modern devices can provide a 360° FOV by adding only a little mass (less than 150 g) and volume (less than 0.16 L) (Insta360, 2022). Ultra-high-speed cameras can reach up to 1 million frames per second; however, with a rather large mass (about 8 kg), size (about 10 L), and power consumption (about 300 W) (Phantom, 2022). Most camera sensors easily cover the color range of the visible light spectrum, some specialized sensors can detect images even in the infrared or ultraviolet light range.

Latest technologies are event-based cameras, which offer attractive properties compared to traditional cameras: they come with high temporal resolutions (in the order of kHz to MHz), high dynamic ranges, and rather low power consumption (Gallego et al., 2017). Embedded event-camera systems, where the sensor is directly interfaced to a processor, have shown system-level power consumption (i.e., sensing plus processing) of 100 mW and even less. At the die level, most event-based cameras use about 10 mW, and some prototypes achieve less than 10 μW, thus, reaching or even outperforming the values of the human eye. The shortest detectable event of these devices lies in the range of microseconds. Therefore, event-based cameras are used in applications that require high refresh rates and low latencies. Image information from event-based cameras can be fused with signals from depth and RGB perception sensors to obtain accurate depth information (Gallego et al., 2017). Dynamic ranges of event-based cameras are above 120 dB, notably exceeding the 60 dB of high-quality, frame-based cameras, making them able to acquire information from moonlight to daylight, comparable to the performance of the human eye. Chip size is in the range of only 1 cm^2^, not including other hardware components (Gallego et al., 2017). Consequently, with the advent of event-based cameras, we experience artificial vision that is indeed starting to measure up to the human eye.

Lightweight 3D camera systems with time-of-flight or stereoscopic sensors (e.g., Kinect V2 or IntelRealsense D415) have a depth perception uncertainty of around 1.5 mm and a bias of 2–5 mm at a distance of 1 m (Giancola et al., 2018). These sensors have a restricted sensing range of up to a couple of meters, while the sampling rate goes up to 90 Hz (Intel Realsense, 2021). The portable LIDAR sensor Ouster OS34, can measure with a precision of 80 mm at up to 240 m distance, which is clearly outperforming the human depth perception (Ouster, 2021). However, the measurement system does not allow simultaneous measurement in the whole field of view and the update rate for a single line of sight is limited, e.g., to 20 Hz for the Ouster OS2.

### Tactile, proprioceptive and vestibular systems

Human density of tactile receptors varies among different areas of the body and is highest on the tongue. Most relevant is the tactile perception on the fingertips as the fingers are important for manipulation tasks. At the fingertips, humans can perceive a spatial resolution of 1.8 mm, a sensitivity of 0.2 g/mm^2^, response threshold range of 0–100 g/mm^2^, and a frequency response of up to 100 Hz (Dargahi and Najarian, 2004). Assuming signal propagation speeds between 2 m/s and 100 m/s and nerve lengths of maximally 1 m, signal latencies are about 10–500 m (Dargahi and Najarian, 2004). The complete coverage of the skin with tactile sensors allows the human to perceive and interact with the environment in a highly versatile way by including arbitrary body parts in interactions with the environment.

The latest artificial pressure sensor matrices can outperform all these biological values easily. Sensing devices reach localization accuracies of up to 0.1 mm at a sampling frequency of 100 Hz (Yan et al., 2021). Some of them are inspired by the human vibrotactile sensing principle (Chun et al., 2021; Dahiya et al., 2009). Visual-tactile sensors were successfully used to detect slip (Yuan et al., 2015; Xia et al., 2022). Li et al., present a comprehensive recent review of the state of the art in tactile perception and application (Li et al., 2020a). The progress in tactile sensor developments leads to increased functionality and performance for robotic manipulation (Kappassov et al., 2015; Luo et al., 2017; Yin et al., 2021; Xia et al., 2022).

Standard kinematic sensors can measure angles and distances proprioceptively in the range of angular seconds and micrometers, respectively (Azibil Corporation, 2023). Inertial measurement units can measure angles with 0.6° accuracy and 0.1° precision (Leah et al., 2017) while sensor fusion approaches combine the high precision and accuracy of proprioceptive, and visual sensors with the robustness and reliability of vestibular sensors (Wisth et al., 2023). Thus, they easily match or excel the performance of the human proprioceptive or vestibular systems. For example, conventional position sensors and inertial measurement units provide high quality signals about posture, movement, and balance. Technical systems are rather compact and lightweight, and do not need a lot of power, so that they can be easily implemented in humanoid robots.

Next to tactile sensing, also proprioceptive force perception plays an essential role for humans to control haptic interactions with the environment. Although the human body does not have any receptors that detect force directly, it can sense different amounts of forces or weights by integrating tactile and proprioceptive receptor information. Proprioceptive force perception is used to robustly track interaction forces, e.g., when polishing objects, dancing with a partner, or steering a vehicle. Similarly like in humans, force perception can be realized using the intrinsic properties of artificial actuators, e.g., with series-elastic or quasi-direct electric motor actuation comparable or even more accurate perception of joint loads to humans can be achieved. More accurately, forces and torques can also be measured by commercially available force/torque sensors that function on resistive, capacitive or optical measurement principles. Typical force sensors used in robotics can have accuracies better than 1% of the measurement range (Cao et al., 2021). They are easily outperforming the human sense.

### Actuation systems

The human muscle is a powerful and efficient organ that can produce high muscle forces requiring little energy supply (see Table 2). For example, the biceps brachii muscle of an average adult human produces a continuous mechanical power output of 50 W/kg (Hunter and Lafontaine, 1992; Hunter et al., 1991a; Huxley, 1980) and peak power to mass values of 100–400 W/kg (Hunter and Lafontaine, 1992; Josephson, 1985; Hill, 1922; Lupton, 1922). Mammalian muscle force normalized by cross-sectional area as a denominator for size can reach values up to 0.35 MPa for peak forces and about 0.1 MPa for sustainable muscle force (Hunter and Lafontaine, 1992; Huxley, 1980). Thus, the sustainable muscle power and force is about 30% (Schoenfeld et al., 2021) to 40% (Leng et al., 2021) or less of the peak value. Mechanical output power in relation to power consumption yields an efficiency of 40% for the biceps muscle (Leng et al., 2021) which is similar for other mammals (Curtin et al., 2018), as work is produced not only mechanically but also metabolically resulting in heat. A specialty of mammalian muscles is their capability to regenerate after injuries and to adapt their dynamic properties (speed, amount of force, fatigue behavior) to slowly varying conditions. The deformable nature of muscles allows a tight packaging spanning one or multiple joints, such as at the shoulder or vertebrae. Further, the series-elasticity introduced by the tendons as well as the antagonistic system allows storage of mechanical energy and selective stiffening up of a joint. For instance, the Achilles tendon can store energy during the stance phase that is released during toe push-off by leveraging the remarkable eccentric strengths of muscles. Moreover, the structure and selective recruitment of muscles enables the impressive versatility from highly delicate interaction to application of large forces and high power.

**TABLE 2:** Actuator Principles. Comparison of different actuation technologies in power density, efficiency (excluding transmission), and force density.

Actuator technology	Working principle	Power/mass	Force/cross-sectional area	Efficiency	Ref
Human Muscle	Biological muscle fibers (contracting actin and myosin filaments)	50 W/kg (sustainable)	0.1 MPa (sustainable)	40%	Hunter and Lafontaine (1992); Hunter et al. (1991a); Huxley (1980); Josephson (1985); Hill (1922); Lupton (1922)
		100–400 W/kg (peak)	0.35 MPa (peak)		
SMA	Shape memory effect of metallic alloys	50 kW/kg	200 MPa	1%–10%	Hunter et al. (1991b); Liang et al. (2020)
EAP	Electroactive polymers	100–600 W/kg	0.2–100 MPa	25%–80%	Youn et al. (2020)
Pneumatic cylinders	Air pressure used to actuate a piston of a cylinder	350 W/kg	0.7–1.2 MPa	4%	Chou and Hannaford (1996); Liang et al. (2020)
PAM	Pneumatic artificial muscles	350 W/kg	0.7–1.2 MPa	49%	Chou and Hannaford (1996); Liang et al. (2020)
Hydraulic cylinders	Oil pressure used to actuate a piston in a cylinder	817 W/Kg	20–30 MPa (comparable size to human muscle) 70 MPa (heavy duty)	53%	Barasuol et al. (2018)
Electromagnetic actuators	Electromagnetic fields used to actuate metallic coils or magnets	312 W/kg	Depends on adopted transmission	96%	Madden (2007); Burt et al. (2008)

Most humanoid robots are equipped with electromechanical motors, while for instance Atlas is actuated by hydraulics. Further actuation technologies applied in robotics are pneumatic actuators, such as pneumatic artificial muscles (PAMs such as McKibben actuators), shape memory alloys (SMAs), and electroactive polymers (EAPs). EAPs include dielectric elastomer actuators (DEAs) and relaxor ferroelectric polymers (RFPs). The latest devices also include ionic polymer metal composites (IPMCs) or hydraulically amplified self-healing electrostatic (HASEL) actuators.

Depending on the type and ratio of the accompanying transmission, electromagnetic actuators, also called electromechanical motors, can produce quasi-arbitrarily high forces. Power density values range from 312 W/kg, when using rare earth magnets, and twice that when they are actively cooled (Madden, 2007). Power efficiencies of motors can achieve up to 96% (Burt et al., 2008). Thus, modern electromagnetic actuators have a slightly higher power density compared to humans.

High-pressure hydraulic cylinders for heavy-duty applications, e.g., the RC10010 (by Enerpac Ltd., United States) can be operated with 70 MPa. However, smaller cylinders and valves built for mobile robotic applications in a scale that is comparable to humans, are typically rated for operation with 20 MPa–30 MPa (Barasuol et al., 2018), e.g., Model E081-115 by Moog uses 21 MPa according to the company data sheet. When considering the outer cylinder diameter, the force per area can be substantially lower, e.g., by a factor of 0.53 for RC1514 (by Enerpac Ltd., United States). Thereby, this actuator achieves a power density of 817 W/kg based on the company data sheet and when using the weight reported for the academic equivalent ISA V5 (Barasuol et al., 2018). Thus, hydraulic cylinders achieve force densities that are two magnitudes higher than human muscles are capable of and around double the power density. MPa Pneumatic cylinders and PAMs can generate forces in the range of 0.7 MPa–1.2 MPa and about 3.5-times higher power densities compared to the human muscle (Chou and Hannaford, 1996; Liang et al., 2020). Efficiencies of PAMs are up to 49%, whereas pneumatic cylinders are below 4% (Chou and Hannaford, 1996). However, these numbers found for hydraulic and pneumatic actuators do not include the weight of the means for pressure generation and storage.

SMAs produce forces of 200 MPa and power densities of 50 kW/kg; with an efficiency of only about 1%–10% (Hunter et al., 1991b; Liang et al., 2020). EAPs generate very high force densities of 0.2 MPa–100 MPa (Youn et al., 2020). Power densities are between 100 to W/kg600 W/kg and efficiencies between 25% and 80% (Youn et al., 2020; Higueras-Ruiz et al., 2021). HASEL actuators produce comparable force densities 0.3 MPa and higher power densities and 614 W/kg, but smaller efficiencies when compared to human muscle (Acome et al., 2018; Liang et al., 2020). IPMC actuators produce higher force densities, but smaller power densities and efficiencies when compared to human muscle (Kim and Shahinpoor, 2003; Liang et al., 2020). So far, these actuation principles are mainly used in nano and micro-robotics, because of their good performance at small scale. Successful application in human-scaled multi-DOF robots was not yet demonstrated.

Combustion machines are rarely used in robotics (and were excluded for the use in human-centered domains in this study), although the continuous power density is about 10 times greater than the continuous power output of muscles (Madden, 2007) and the forces can be high using transmissions with high gear ratios. Furthermore, gasoline has a high energy per unit mass that is about 20 times higher than that of a good battery, even after accounting for the approximately 30% efficiency that is typical in combustion processes.

All these actuation technologies (except for IPMCs) outperform human skeletal muscles with respect to force and power densities, when neglecting the weight of energy storage and pressure generation. Besides pneumatic cylinders and SMAs, they are also providing higher power efficiency.

Most artificial actuators outperform the human muscle concerning speed and endurance because the generation of muscular force is bound to rather slow electrochemical processes and the force levels cannot be kept constant over long time periods due to muscular fatigue.

Efficiency values were obtained primarily from Higueras-Ruiz et al. (2021); they consider the total system including off-board energy storage to output work. However, the force and power density data of the technical actuator systems do not include components that store and provide electric, fluidic, or thermal power (e.g., battery, compressor, pressure tank, heating system, respectively). They also do not include components that are required to change actuator speeds (e.g., transmissions and clutches), that which take away the heat (e.g., air ventilation or liquid cooling systems), or any other components such as sensors, cables, electric, and thermic or acoustic isolations. As many of those components can be rather bulky and heavy, they would weaken the force and power density values, which likely gives the human actuator an advantage over artificial ones. Unfortunately, the weight of those auxiliary components is largely unreported in the literature; and it varies a great deal between different solutions and applications. Other studies investigating methods for embodied energy strive towards more lightweight and untethered energy supply and conversion methods (Aubin et al., 2022). For most of such concepts, demonstrations were either performed at a very small, micro-robotic scale, on isolated actuator systems, or on robots with the primary purpose to showcase the actuation method.

Series-elastic actuation is frequently used in walking systems (Hutter et al., 2016a) or in robots that are in interaction with humans (Vallery et al., 2008; Zimmermann et al., 2019). The energy-storing and release capability of these actuators was often envisioned, however, so far barely successfully exploited by state-of-the-art systems. Series-elastic actuators have further advantages that can be nicely exploited in robotic applications, e.g., the ability to withstand shocks due to mechanical compliance. However, the design and control of robots with such actuators is challenging, because of the additional mechanical components that lead to higher compliance. Many demonstrators with novel series-elastic actuators including variable impedance control exist in the scientific literature (Ham et al., 2009; Vanderborght et al., 2013; Verstraten et al., 2016). There have been a number of approaches for tendon-driven series elastic actuation approaches for bipedal humanoid robot locomotion aiming for utilization of the passive synchronization properties of the mono and biarticular muscle groups like in the human leg (Seyfarth et al., 2009; Sharbafi et al., 2016; Sharbafi et al., 2016).

Systems realizing more efficient use of materials by embedded energy storage (i.e., combining energy storage with actuation) were demonstrated in less versatile robots applied to less complex cases than humanoid robots (Aubin et al., 2022). Soft actuation principles promise to close the gap in power density and more optimized system integration due to the flexible structures. However, these technologies are to date still in an early phase where prototypes are mostly investigated in isolated settings and not incorporated in full robotic systems (Rothemund et al., 2021).

### Skeletal systems

The skeletal systems of the human and robot are comprised by links (i.e., bones) and joints that connect the links allowing relative movement. Human bones can transfer rather high mechanical loads and have impressive self-healing capabilities and other relevant functions, such as blood cell production. Most joints in the human body are synovial joints. Ligaments, tendons, and other connective tissues form the articular capsule with a synovial cavity between the bones. Synovial joints are self-lubricating, almost frictionless, and able to withstand heavy loads in the magnitude of the body weight (and higher), while still executing smooth and precise movements. Human synovial joints are difficult to outperform by artificial joints (Table 3). Only very dedicated pairs of materials or ball bearings can compete with the friction properties of human joints. In contrast, robotic joints can have larger ranges of motion, even up to 360° or more, which is not possible with physiological joints.

**TABLE 3:** Friction coefficients of different joints and pairs of materials and a human joint (Serway and Vuille, 2014; Sagbas, 2016; Koyo Bearings/JTEKT CORPORATION, 2022; Engineering ToolBox, 2022b; Copper, 2022).

Joint or pair of artificial materials	Friction coefficient
Human joint	0.003
Ball bearings	0.002
Steel on steel (lubricated)	0.16
Steel on bronze (clean and dry)	0.08–0.14
Artificial hip joint (metal on polymer)	0.06

The adult human body comprises 206 bones that are connected by joints. Each of these joints has negligible, one, or multiple degrees of freedom. The number of degrees of freedom per joint depends on the complexity of the kinematic model. For example, the knee joint not only flexes in the sagittal plane of the body, but it can also move outside the sagittal plane (“varus-valgus”) or rotate around its longitudinal axes. Due to the mechanical compliance of the joint capsule and cartilage, it can even move in translational directions. Thus, knee joint models can vary between one and six DOF. Even simpler models count about 155 DOF (Venture et al., 2007) in the human skeleton. This high number of DOF enables a large range of motion at certain body regions such as the shoulder or the trunk, allowing manyfold, dexterous grips of the hand, and generates vast kinematic redundancy of the extremities when interacting with objects, humans, or other robots. The most advanced humanoid robots are counting maximally only one-third of the DOF of the human skeleton (Table 4).

**TABLE 4:** The number of degrees of freedom (DOF) of different robots in comparison to a simplified model of the human (Takiyama et al., 2020; Yamane et al., 2005; Li, 2006; ROBOTIS, 2022; ikinamo, 2009b; Samson et al., 2001b; Hutter et al., 2016b).

System	Degrees of freedom (DOFs)
Human	155 (model)
Asimo	57
HUBO	32
iCub	53
NAO	25
Atlas	28

**FIGURE 2:**
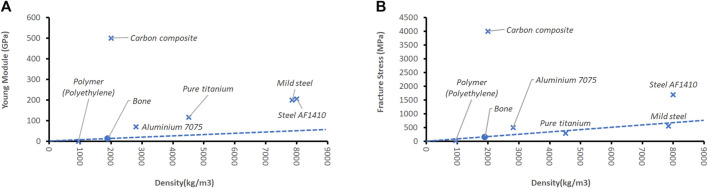
Two features to describe the properties of bones are elasticity expressed by the Young Module **(A)** and Fracture Stress **(B)**. Features are normalized by the denominator bone density, which correlates to the mass of the material. (The world material, 2022; Cameron et al., 2000; HABA, 2021; Garrison, 1990; Cao et al., 2009; HABA, 2021; Garrison, 1990; Reed and Brown, 2001). The dotted line indicates the linear extrapolation of the normalized average human performance.

In robots, bones are represented by rigid links (bars) made of metal, polymers, or carbon composites. Many metals and carbon composites do have better stiffness and fracture properties than human bones (Figure 2).

## Comparison of functions

### Walking

Normal walking is one of the most developed functions of humanoid robots. The preferred natural walking speed of humans is about 1.4 m/s (Samson et al., 2001a). We compared the natural human walking speed with those of the 22 bipedal robots found in this study. Walking speed has been related to the denominators mass, size, power consumption and inverse of the operational time.

**FIGURE 3:**
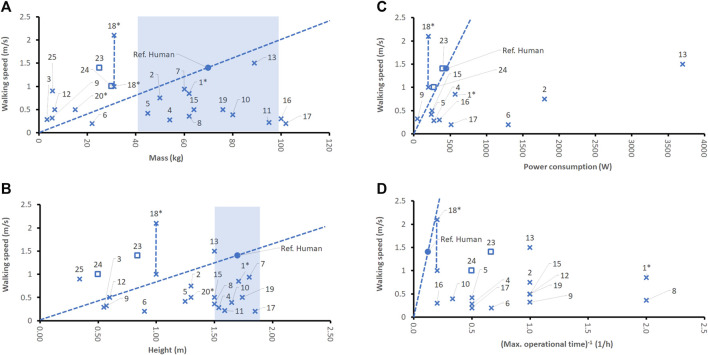
Comparison of robots’ walking speeds for bipeds (x), quadrupeds (□), and legged robots with rolls (o) with respect to denominators mass **(A)**, height **(B)**, power consumption **(C)**, and the inverse of the maximum operational time **(D)**. Bipeds without humanoid upper bodies (without torso, or without functional hands or head) are marked by a star (x*). Maximum operational time and power consumption refer to common activities, such as walking. Note that not all robot sources provided data about all features studied. For the human test case, the so-called “preferred walking speed” (Samson et al., 2001a) was chosen. For Cassie (no. 18) two different walking speeds have been reported in the literature. Both are displayed and connected by a vertical line. The dotted line indicates the linear extrapolation of the normalized average human performance. The blue shade indicates a typical range of the denominator for humans (2^nd^ percentile female to 98th percentile male).

When relating natural human walking speed to mass or size (Figures 3A, B), one can see that many of the robots cannot compete with humans, because their walking speeds in relation to their physical dimensions (expressed by robot mass and robot height) are lower than that of humans. In contrast, Cassie clearly outperformed the human test case with respect to natural gait stepping speed. However, Cassie does not have a torso nor arms and head, giving it an advantage with respect to its mass. The quadruped robots Spot, ANYmal, and Titan clearly outperformed the human test case with respect to natural gait stepping speed. Other robots, such as HR 18, NAO, QRIO, Asimo, LOLA, and Atlas are showing similar walking speed performances to humans. Flame performs about as well as humans when relating gait speed to its mass, but not when relating it to its height, because this robot is rather “skinny,” thus, lightweight, and tall (only around 16 kg at heights of more than 1.2 m). From a pure kinematic point of view, a tall robot has an easier job performing large steps leading to a higher gait speed. Also, when relating natural walking speed to power consumption, not many robots can outperform humans (Figures 3C). Human power consumption during walking was assumed to be 450 W. Only Cassie, NAO and Robotis are outperforming human walking speed when relating it to power consumption. All these robots have very small masses and heights, making it obviously easier to realize an efficient energy concept. Cassie, a robot without even a torso and one of the newest bipedal robots, reaches a maximum walking speed of 2.1 m/s (Michigan Robotics, 2020); it consumes about 200 W, when walking with a speed of 1 m/s (Kashiri et al., 2018). In contrast, Atlas requires more than ten times more power to reach about the same walking speed as Cassie. Digit (C et al., 2021; NED Directory, 2023; Robots, 2019), the successor of Cassie, has a maximum walking speed of only 1.5 m/s. Furthermore, Digit is heavier than Cassie (42.2 kg–48 kg, depending on source, vs. 31 kg) and taller than Cassie (1.55–1.58 m vs. 1.15 m), which worsens the robot’s features when normalizing them with respect to denominators mass and size. Digit’s run time is equal to the one of Cassie (light duty run time: 3 h, heavy duty run time: 1.5 h). It seems that Digit’s additional trunk and arms (despite the great additional functions) reduces its performance in our metrics compared to Cassie.

Instead of power consumption, also Cost of Transport (CoT), can be used to normalize walking speed and compare it among different robots and the human. CoT, also called “specific resistance,” expresses power consumption, and relates it to mass and locomotion speed. The CoT of biological systems is far superior to those of robots (see Table 5) The reported CoT values of humans and robots are sample values taken from the mentioned references. The CoT is a function of the gait type, walking speed conditions, the kinematic characteristics of the individual, etc. Thus, reported CoT might vary between references.

**TABLE 5:** Comparison of Cost of Transport (CoT) and power consumption of different robots for walking.

Number	Name	Type	References	Power consumption (in W)	CoT
	References human	Biped	Tucker (1975)	450	0.38^1^ to 0.47^2^
2	Asimo	Biped	ASIMO (2022)	1800	2
16	Durus	Biped	Wevolver (2022)	350	1.5
17	Walk-Man	Biped	Thomson Reuters (2015); Ackerman and Guizzo (2021); EurekAlert (2018); Instituto Italiano di Technologia (2018)	510	1.35
18	Cassie	Biped	Xie et al. (2019); Gong et al. (2019); IEEE Spectrum ROBOTS (2022a)	200	0.7

Results are presented only for those robots where data has been found in the literature or robot data sheets. The human values are repored for walking fast (^1^) and jogging (^2^).

The data found on operational time refers to normal usage conditions, and not any extreme condition. All robots functioning with an autonomous energy supply (i.e., portable batteries) exhibit performance inferior to the human test case when relating their walking speed to the operational time (Figures 3D). Although robots such as Cassie, and Atlas are reaching rather high walking speeds, their operational time is significantly lower compared to humans.

### Running

Running is defined as a legged locomotion pattern that includes a short flight phase in which both feet do not touch the ground. Running can be characterized by running speed and endurance. In the human-centered domain, there is a high variability of performance among individuals. The world’s best athletes can finish a marathon distance at an average pace of about 21 km/h or run 160 km with 10 km elevation gain in little more than 1 day (26.4 h) (Swiss Alps 100, 2021; World Athletics, 2022). For an average human, we estimate the operational time for running to be at least 1 h. Assuming an average jogging performance, a healthy young adult can run at an average pace of 3.06 m/s for quite long times (Barreira et al., 2010), with an optimal, energy-efficient running speed of 3.7 m/s for males (Steudel-Numbers and Wall-Scheffler, 2009).

**FIGURE 4:**
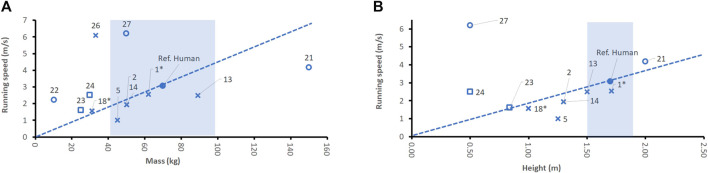
Comparison of running speeds for bipeds (x) and quadrupeds (□) with respect to denominators mass **(A)** and height **(B)**. Biped robot ATRIAS (no. 1) has no arms and no head and is, therefore, marked by x*. Note that not all robot sources provided data about all features studied. The running speed of the human refers to a human jogger running at a pace of 3.06 m/s (Barreira et al., 2010). The dotted line indicates the linear extrapolation of the normalized average human performance. The blue shade indicates a typical range of the denominator for humans (2^nd^ percentile female to 98th percentile male).

Also, some legged robots can perform a running pattern. One of the newest and best performing humanoid robots, Atlas, has a battery cycle time of about 1 h for running, and reaches maximum running speed of only 2.5 m/s, which is inferior to the human. Robots ATRIAS, Asimo, and Toyota Partner are jogging with a similar performance to humans, when referring to their mass and size (Figure 4). For most robots, there is no data available about running endurance and power consumption.

The COT of MIT cheetah (0.52) came closest of all robots to the COT of a human (0.2–0.47). Further, MIT cheetah outperforms a jogging human: it runs at a pace of 6.1 m/s and weighs only 33 kg (Figures 4A). Obviously, four legs enable much faster running speeds than two legs. Its drawbacks, however, are that its power consumption is high [973 W (Seok et al., 2014)] and operational time cannot compete with that of a jogging human. However, cheetah did not achieve this performance while transporting means for manipulation.

Adding trunk and arms for functions other than locomotion, such as perception and manipulation, would further downgrade running performance of the robots. One of the reasons, why it is so difficult to outperform human running performance could be the fact that mammals like the human comprise elastic components, tendons, that store and release potential energy and compensate shocks when the foot touches the ground. This makes the running very energy efficient and keeps the foot on the ground and avoids too much shaking of the other parts of the body. Most humanoid robots do not contain such components yet.

Legged robots rolling on wheels can outperform humans when referring the feature running speed to the denominators mass and/or size. Some recent examples for such robots are Handle and the version of ANYmal equipped with wheels connected to its feet (Bjelonic et al., 2020; IEEE Spectrum Robots, 2021), Ehile the idea of combining wheels weigh legs is much older and shares a blurry border with active suspensions (Smith et al., 2006). ANYmal on wheels achieves a locomotion speed of 6.2 m/s using the wheels for propulsion (Swiss Mile, 2022). However, comparing the running speed of an average human might not be a fair comparison, as the human could also use rolling means of transport (e.g., roller blades).

### Stair climbing

**FIGURE 5:**
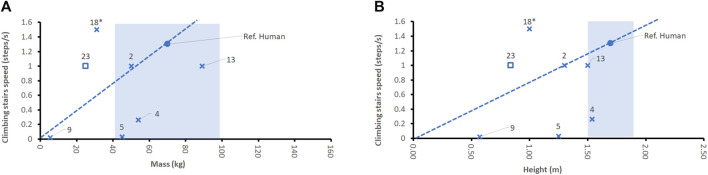
Comparison of robots’ stair climbing speeds for bipeds (x) and quadruped Spot (□, no. 23) with respect to denominators mass **(A)** and height **(B)**. Cassie (no. 18) has no torso and no arms and head and is, therefore, marked by x*. Note that not all robot sources provided data about all features studied. The dotted line indicates the linear extrapolation of the normalized average human performance. The blue shade indicates a typical range of the denominator for humans (2^nd^ percentile female to 98th percentile male).

The average stair climbing speed of an adult human male or female is about 0.66 m/s or 0.48 m/s, respectively (Choi et al., 2014), which corresponds to an average of 1.3 steps per second (Hinman et al., 2014). Also, most legged robots can ascend and descend stairs. The stepping speed of the robots has been estimated from public video material and displayed in relation to mass or height (Figure 5), see Supplementary Appendix, as there was no quantitative information available in the literature or in the data sheets of the devices.

Except NAO, all the stepping robots considered in this paper can overcome step sizes that are comparable to steps in human-centered domains, or even higher. Asimo and Atlas perform stepping speeds that are comparable to the human test case. These two robots have body masses and heights that are like those of an average human. Only Cassie and Spot outperform a human, as they can reach about the same numbers of steps per second, as a human, at human-sized steps. However, Cassie has a body mass that is less than half of an average human. It seems to have again an advantage, thanks to its missing torso and arms. However, this prevents its application for other tasks such as carrying and manipulating objects. And operational time is probably lower than that of a human walking.

A company video (Smith Marshal and Marshal Smith Technology, 2018) shows quadrupedal robot Spot climbing a normally sized staircase in a human laboratory environment. The robot climbs 7 steps in about 7 s, thus, achieving a stair climbing speed of 1 steps/s, see Figure 4.

### Changing and maintaining body postures

There are robots that are designed to specialize in mimicking human gestures, e.g., the RASA (Meghdari et al., 2019). Other devices can manipulate their environment, while optimizing the posture for improved load transfer in confined workspaces (The Verge, 2015). These robots perform well in tracking precise end-effector trajectories if the attached load is constant. However, for tasks involving heavy interaction, e.g., cutting with power tools or determining precise and accurate position trajectory, tracking is a challenge (Gawel et al., 2019; The Verge, 2015). Further relevant human body functions listed by the International Classification of Functioning, Disability and Health (ICF) are lying down, squatting, kneeling, sitting, standing, bending, and shifting the body’s center of gravity (World Health Organisation, 2007). Those functions can be performed by some, but not many humanoid robots (Tektronics Expert, 2016; Fakultät für Elektrotechnik und Informationstechnik, 2022; Romualdi et al., 2018; Beira et al., 2006; Pontes, 2019). iCub, for example, is instrumented with very precise position and inertial sensors. Consequently, iCub can track trajectories and perform its postures with a very high repetition accuracy, perhaps comparable to those of Tai-Chi athletes (Poppy Mosbacher, 2020). Thus, iCub outperforms the average human with respect to posture accuracy, as many other robots do, even if their repertoire of implemented postures is much smaller. Furthermore, robots have a better postural endurance than humans. The ability to control body postures in a humanoid robot (as well as posturally stable walking and running) is closely related to two key functions: the robot’s artificial vestibular sensing system (i.e., an inertial measurement unit providing sufficiently fast and accurate 3D linear acceleration and 3D rotational angular velocities, and/or visual sensors) and the actuators being able to perform sufficiently fast enough motions. In addition to the actuation system (e.g., electric motor), brake systems can be deployed that use form fits or friction to lock the robot joint’s. While these systems require only little to no power to hold the position, they add weight and volume to the system and consume energy to change the state. Dexterity and velocity in performing such postures and movements, however, remain superior in humans.

### Other movement functions: negotiating rough terrain, crawling, jumping, swimming

Comparing capable robots with the average human can provide valuable information on the agility of these robots in rough natural terrains. The very dexterous platform, Atlas, can jump as high as an adult human, do backflips, and negotiate highly challenging artificial terrains (Boston Dynamics Support Center, 2021). Crawling on the ground can be performed by the small humanoid, iCub. With an estimated crawling speed of 0.03 m/s (i. HumanoidRobot, 2010) it is relatively slow with a body height of 1 m compared to humans crawling up to 1.47 m/s (Gallagher et al., 2011). The very specialized humanoid robot Swumanoid (Plastic Pals, 2022) can perform a crawl swim at 0.12 m/s (Nakashima and Kuwahara, 2016), while average human swimmers reach 0.96 m/s (Seifert et al., 2010). The bipedal rolling robot Handle can jump up to 1.2 m (Boston Dynamics, 2017; IEEE Spectrum Robots, 2021). Quadrupedal walking robots like ANYmal and Spot recently have shown great performance in negotiating rough terrain robustly. ANYmal was shown to negotiate rough terrain matching the speed expected for an average human on a 2.2 km hike and 120 m elevation gain in a total time of 78 min, while being lighter than a human (Miki et al., 2022). Further, the ANYmal robot demonstrated robust performance in narrow subterranean tunnels (Tranzatto et al., 2022).

### Driving

The DARPA Robotics Challenge required the participating humanoids to drive a small vehicle a short distance by interacting with a steering wheel and one pedal. Many platforms have consequently been programmed to drive autonomously or teleoperated. As shown by Goswami and Vadakkepat (2019) the robot HRP2 could successfully perform this task, by driving a small buggy. A similar performance was observed by IIT robot Walk-Man (Instituto Italiano di Technologia, 2015) and other robots that participated at the DARPA Challenge. However, the vehicle was specially designed for this challenge allowing simplified access and control, and the driving was relatively slow. The dexterity of the driving robot was far inferior to that of an average human driver (Signe Brewster, 2015).

### Lifting and carrying objects

The Swiss State Secretariat for Economic Affairs outlines the maximum allowed loads carried by (human) workers, depending on their age and gender, and ranging from 10 kg to 25 kg (Swiss State Secretariat for Economic Affairs, 2016). However, the weights carried by a well-trained human can be much higher. The robot, Atlas can successfully lift and carry 5 kg boxes while the payload to Toro was 10 kg. The Handle robot can carry weights of up to 45 kg, but only when it is rolling, not stepping (Boston Dynamics, 2017; IEEE Spectrum Robots, 2021).

### Kicking objects with legs and playing soccer

In the Humanoid League of the RoboCup, autonomous robots with human-like bodies and senses play soccer against each other in a competition. Unlike robots outside of the Humanoid League, walking, running, kicking the ball, balancing, visual perception, self-localization, localization of the overall soccer scenario, team play, strategy planning etc. must be human-like. Several of the best autonomous humanoid robots in the world, such as NAO, compete in this competition. While these humanoid robots fulfill structural demands, such as the right anatomy to trap and shoot the ball and locomote between other (robotic) soccer players, dexterous motor functions, such as dribbling, sensory functions, such as an accurate localization of ball and other players, and higher cognitive functions, such as strategy planning, are still lacking. Consequently, soccer capabilities, especially game speed and dexterity, are still far below that of human soccer players. When establishing the RoboCup initiative in the nineties of the last century, the organizers envisioned to build humanoid robots that are capable to beat the human world champion team playing according to the FIFA rules in 2050 (Gerndt et al., 2015).

### Manipulation

The human hand has approximately 27 degrees of freedom (DOF), with 6 actuators and 4 DOF for each finger (Goswami and Vadakkepat, 2019). The spatial accuracy of the human hand of a surgeon can reach values of less than 1 mm (Kwartowitz et al., 2007; Choi et al., 2009), while the same hand is capable of generating fist forces of more than 484 N (male, mean + 1SD) (Hogrel et al., 2007). Humans are highly capable of exploring and manipulating objects due to their efficient sensory and motor functions. Human sensation employs sensor fusion of tactile, proprioceptive, visual, and auditory modalities with high spatial and temporal resolution, leading to rich information required to interact with the environment. Furthermore, human muscles are actuated in a highly selective way with respect to space and time, due to the presence of many small motor units that can be recruited independently. Therefore, humans are highly efficient in bimanual manipulation and task execution. One of the most agile robotic hands integrated into a humanoid, is the one in iCub, which has 9 DOF in total. Humanoid hands that are detached from a robotic trunk and mounted to a rigid frame can have a higher number of DOF (Szkopek and Redlarski, 2019). For example, the Gifu Hand III has 16 DOF (Kawasaki and Mouri, 2019), and the Shadow Hand even 20 DOF (Rothling et al.). Recently published review articles present the current state-of-the-art and future trends of robotic grasping and manipulation, including sensing, actuation, and control (Billard and Kragic, 2019; Cui and Trinkle, 2021; Sleiman et al., 2019; Yin et al., 2021).

Purpose-built robots such as the surgical tele-manipulator DaVinci achieve accuracies of about 1 mm (Kwartowitz et al., 2007) in dexterous multi-DOF robotic manipulation. Thus, they can compete with human surgeons regarding accuracy. However, the DaVinci has a fixed base, is 175 cm tall, and has a mass of 545 kg (intuitive, 2022). Similarly, robots built for pick-and-place excel human performance in speed [e.g., YF003N (Kawasaki, 2023)]. However, these robots do not meet our inclusion criteria regarding mobility.

Human-centered domains challenge robots with a large variety of shapes, softness, and surface properties of objects to be manipulated. To cope with this large variety of objects, novel machine learning methods applied to sensor fusion and control seem to be promising (Cui and Trinkle, 2021). Rakita et al. (2019) introduced a method to transfer human bimanual manipulation strategies to a humanoid robot. A shared-control policy allows the user to telemanipulate the robot, while being supported by an assistance controller that identifies typical patterns from a bimanual action library in the human commands (Rakita et al., 2019). Understanding the tactile perception of shapes, stiffnesses, and contact states (e.g., slip) is crucial to advance manipulation capabilities in robots. The recent developments in visual perception processing improved vision-based object localization, tracking and semantic characterization. Many of the methods developed for visual perception also find their applications in the processing of tactile sensor signals (Luo et al., 2017). The fusion of tactile, visual, and proprioceptive sensing modalities leads to versatile and robust object and contact state perception (Xia et al., 2022). Li et al. (2020b) demonstrated the use of a multi-modal tactile sensor to classify objects based on pressure, temperature, and thermal conductivity information. This progresses also advanced the manipulation of deformable objects, which is still one of the biggest challenges for robot manipulation (Yin et al., 2021). Because of the highly efficient sensory and motor functions in humans, robots are still performing inferior in dexterous manipulation tasks, particularly when complex tactile perception is demanded. Some robotic systems that are capable of choosing and connecting different robotic hands to their most distal limb to allow a higher versatility of tasks, e.g., to switch between precision and power grip (Billard and Kragic, 2019).

Despite the recent advancements in the development of robotic hands and arms for mobile robots, mobile robotic manipulation lacks far behind average human skills. One eminent difference is the lack of versatility in robotic hands preventing them from performing both precise finger movements as well as powerful grasping and manipulation tasks using a large number of DOF. Next to the mechanical challenges, also controls and perception pose challenges to mobile robotic manipulation. Arguably, the biggest discrepancy between human and mobile robotic manipulation is the semantic and physical understanding of the environment the robot is interacting with (Billard and Kragic, 2019). Likely this is why work environments for robots are still structured to allow robots that are less versatile than humans to successfully perform manipulative tasks.

### Basic motor learning

Motor learning performance is difficult to quantify in numbers. Human and machine learning follow different paradigms but are becoming more and more comparable as the performances of CPUs and artificial intelligence algorithms increase. Human motor learning follows a learning curve that is estimated to lead to acceptable performances within 20 h of practice; however, more than 10′000 h are required to be fully proficient in a complex motor skill, such as surgery (Hirschl, 2015; Kaufman, 2013). Learning algorithms of machines can be much faster in reinforcement learning of motor abilities than humans as multiple scenarios can be evaluated in parallel physics simulations (Miki et al., 2022; Hwangbo et al., 2019).

## Discussion

### Robotic vs. human structures

Artificial vision sensors based on traditional CCD and CMOS chip technology can outperform the eye in temporal and spatial resolutions. However, comparisons must be interpreted with care, because biological and artificial vision systems are based on different design principles and functional paradigms. While digital cameras work with discrete framerate and pixel numbers, the human eye follows the laws of neural activation, resulting in a fusion of continuous stimulations (Scharnowski et al., 2007). Human vision works with higher-level interpretations and sensor fusion to augment the perceived image information.

CCD and CMOS based digital camera systems are inferior to the eye in other features: they have smaller dynamic ranges and higher dark limits than the human eye, and the denominators mass, size, and power consumption are larger. Specialized RGB sensors can outperform the eye, when dealing with non-visible light or in very dark environments, where the human eyes do not perform at all. However, these specialized sensors tend to trail behind nature regarding other specifications.

At the cost of relatively high mass and volume, LIDAR sensors can perceive depth with high accuracy, clearly superior to human eyesight. However, due to the measurement principle, the latency of measurement updates for a field of view is higher, thus inferior to the human eye. Therefore, structured light and stereoscopic sensor technologies, which are characterized by low latency, but less accurate depth perception, are often used in combination with LIDAR sensors for the analysis of the environment close to the robot where fast reactions are needed.

The latest event camera technologies do finally compete with the performance of the human eye with respect to latency, dynamic range, motion blur, and power consumption. (Gallego et al., 2017). However, there are different trade-offs involved, such as latency vs. power consumption, or sensitivity vs. bandwidth and processing capacity, or camera size vs. spatial resolution. Overall performance could be further improved by pairing an event camera with depth sensor technologies, or an RGB camera to provide 3D depth information and a larger color range, respectively.

Conventional vision systems on board humanoid robots might be sufficient for most human-like tasks in human-centered domains, as for most tasks, optical challenges are not extreme. Furthermore, only one or two vision sensors are required, and not hundreds of units (as for the joints and actuators, so that additional mass, size, and power consumption may not over compromise the entire robotic system. Last, but not least, the visual perception of the environment can be augmented by scans obtained from a set of stationary camera systems and/or cameras mounted on other robots and unmanned aerial vehicles (UAVs) sharing the same domain.

Artificial microphones outperform the human hearing system in terms of perceived frequency range and detection threshold sensitivity. Sound localization accuracy, however, is only better when using larger microphone setups that may not be carried by mobile platforms.

Artificial pressure sensor matrices can outperform human tactile perception. Other sensor technologies outperform proprioceptive and vestibular senses.

The power efficiency of human muscles is better than the one from SMAs and pneumatic cylinders, but most electromechanical and hydraulic systems, PAMs, and EAPs outperform human muscle efficiency, even when considering off-board energy storage. Most artificial actuator technologies outperform the human muscle also with respect to maximum speed and endurance.

It seems that many technical actuator systems also outperform the human muscle with respect to generated force and power density, but only when excluding the mass of power supply and other technical components that are required to provide pressure (compressor, pump, pressure tank/reservoir), heat, or high voltage, thus, a tethered system. Many actuators require heavy active cooling systems. Electromechanical actuators require heavy transmissions to reduce the high rotational speed that they generate (Madden, 2007). Some kinds of EAP technologies require high voltages, which makes handling difficult or even dangerous (Chen et al., 2020). Those auxiliary components need to be carried by the robot, if it is made completely untethered, such as in the hydraulically driven (and heavy) Atlas robot (Ackerman, 2015).

Unlike artificial actuators’ metrics, the metrics for biological muscles account for the weight of auxiliary components like power source (organic compounds, such as Adenosine Triphosphate (ATP), that store and provide energy to drive many cellular processes. Normalization of human and robot actuation including the weight of components that are required to run the actuation can lead to an improvement of the specific metrics of biological muscles compared to artificial actuators (Higueras-Ruiz et al., 2021).

Internal combustion engines seem to be an attractive option for use onboard a robot, because of the high energy density of gasoline. However, according to Madden (Madden, 2007), there are two main drawbacks of combustion engines: First, the engine operates only over a narrow range of speeds requiring heavy transmissions and slip clutches; and second, the machines produce unwanted side-effects such as heat, noise, and fumes. That’s why we have excluded such engines in the frame of this comparative study.

The human skeleton has a much higher number of degrees of freedom than even the most advanced robot. This leads to kinematic redundancies that permit humans to adapt their execution of movements to environmental, object-specific, and task-specific constraints (e.g., moving a filled cup of water in a narrow space). This is one important aspect enabling the enormous task versatility of humans. Furthermore, mammalian tissues such as bones and muscles have regenerative, self-healing, capabilities, and can grow and adapt their properties slowly, if exposed to new conditions in a repetitive manner. In contrast, robotic joints, and links perform at least as well as their human counterparts: the range of motion is much larger in artificial joints, friction properties are comparable, and robotic links made from carbon or special metals, such as titanium, outperform stiffness and fracture properties of the human bone.

### Robotic vs. human functions

Humans evolved over millions of years to be able to perform complex and versatile bodily functions with high levels of dexterity using highly optimized motor, perception, and computational structures. For example, the human sensory-motor function of our nervous system is the product of hundreds of millions of trials and errors of biological interactions with environments which helped creating a highly complex network of neurons that lead to optimized motor control capabilities. In contrast, robot control functions are extremely primitive.

With respect to natural walking speed, only a few robots, including several four-legged robots, outperform human capabilities or get close to them. Most recently developed robots tend to be lightweight and small builds. The power consumption of some of the robots is within a similar range to human power consumption, especially when they have been optimized for walking like Cassie [see Cassie (Xie et al., 2019; Gong et al., 2019; IEEE Spectrum ROBOTS, 2022a)]. Cassie seems to have an advantage because of its low mass due to missing torso, arms, and head. This changes to the disadvantage of the robot when trunk and arms would be added to it, such as Cassie’s successor Digit, which walks slower than Cassie. No robot could outperform humans in terms of gait speed when relating it to cost of transport or operational time.

Some robots can perform fast running patterns, as fast as a human jogger, or even faster, with lower mass and size properties. However, within the scope of this study, there is no robot that runs as fast as a human sprinter, not even the legged robots with wheels. Several robots can climb stairs, but only the best among those are reaching climbing (i.e., ascending) speeds that are comparable to those of an average human when related to body mass or size. All of those robots cannot compete with the human test case when relating their performance to maximally possible operation time.

Few robots can perform a rich repertoire of different body postures–speed of postural changes and dexterity are superior in humans. However, robots have better postural sensors than humans allowing them to perform postures and tracking movements with a higher repetition accuracy, and, depending on actuation and transmission type, remain in a posture virtually forever, without the consumption of additional power.

Other humanoid robots can jump, crawl, or swim like humans, reaching about the performance level of the average human population. Robots can also lift and carry weights, manipulate objects, drive cars, or play soccer, etc. The dexterity and versatility of robotic manipulation improved remarkably within the last decade. In simple pick and place tasks, robots are even much faster than humans, e.g., YF003N [by Kawasaki Robotics, (Kawasaki, 2023)]. However, in dynamic manipulation tasks and handling of deformable materials, robots are still far behind human performance. In general, robot design is still specialized to perform a limited number of specific movement patterns or tasks. Thus, the versatile dexterity of humans is, by far, unmatched by current robotic systems.

The performance of four-legged robots and the commercial availability of such platforms has experienced a steep incline during the last few years. A similar leap in technology can be expected for two-legged robots in the future. However, the design of a robot to perform typical human tasks might not have to strictly mimic the bipedal human structure.

To close the gap between robot and human manipulation capabilities, sensor fusion approaches should be investigated merging full-body, high-resolution sensing of tactile, proprioceptive, visual, and auditory information for environment perception (e.g., semantic information and physical characteristics). To date, most robots still rely on a confined set of contact points limiting the versatile use of their body parts. In contrast, humans perform multi-contact interactions which enable more efficient and versatile manipulations. Future research on more integrated actuation, sensing, and power supply as well as high-resolution soft tactile sensors will pave the way for advanced manipulation capabilities.

Perhaps, the functional limitations of robots can be explained by the fact that human (and animal) anatomy and physiology are based on different principles. For example, humans comprise elastic components that make locomotion functions more efficient and smoother. Control of actuators occurs in different temporal and spatial resolutions in humans *versus* robots. And human use feedforward-control modes to perform movements (e.g., reflexes), whereas robots predominantly use feedback-controlled principles. A more thorough comparison of such anatomical and physiological differences would require another survey paper.

### Limitations

Making a “fair” comparison of technical systems with the human is not trivial, as robots are made by artificial materials and not by biological cells, and robots can have different geometrical and structural appearances, which make it difficult to define a good normative basis. Furthermore, many devices have been developed for a specific application case. Typically, only the specifications relevant to this application case are reported in the scientific literature, making a thorough comparison of devices difficult that depicts the design tradeoffs. Nevertheless, we tried to perform an “apple with apple” comparison, by carefully selecting concrete functions and structures, with their measurable features that are relevant in human-centered domains, and scaling them by meaningful denominators. This scaling was required for a more inclusive review of the existing technology. Assuming a linear relationship of the feature value to the denominator near the human denominator value (i.e., linearization point) allowed to compare the performance of robots with slightly different weight and size to the human performance. However, the assumption of a linear relation between the functional features and denominators could be refined by incorporating known non-linear relationships, e.g., inverse proportionality of power density to weight and volume (Aubin et al., 2022). Alternatively, restricting the comparison to actuation technology that was demonstrated to achieve functional features in the relevant range for the targeted application would allow for a comparison without the linear approximation. Thereby, the tradeoff to overlook technologies with high potential in the near future would have to be accepted. Furthermore, we encourage establishing a unified set of metrics used in publications, such that the performance and design trade-offs of related work can be analyzed more thoroughly.

### Outlook

Humans started early in their evolution (phylogenesis) as well as in their individual development (ontogenesis) to specialize on a subset of specific tasks to optimize the output of their work while relying on social structures to substitute the other tasks. While humans hone the most relevant skills and loose seldomly used skills, the structural parts of the human body persist which inflicts bounds on the performance. Robots built to excel in versatile human functions are not bound to one set of structural components. Thus, the question emerges if one robot design with a versatile functionality or a “society” of robot designs with specialized complementing functions will be favorable.

Even if multiple robot designs collaborate to accomplish the desired functionality, the state-of-the-art in the integration of technical structures to the full robot design would not suffice to provide the functionality with an applicable robot group size. To achieve denser functionality regarding volume and weight more integral combination of the individual components should be strived for as outlined by Aubin et al. (2022) at the example of embodied energy.

Such integral system designs are practically highly challenging to develop, as a high amount of financial and manpower resources would have to be committed to one project. To date, economic and academic structures, and funding favor distributed risk by investing in individual development of modular structures. Thus, in order to develop a highly integrated system with the functional versatility of a human, according financial and sustainable project management structures would be required. To this end, a study investigating the prevalent limitations in versatility of humanoid robotic systems in comparison to highly specialized and repetitive tasks would be of interest.

Disregarding the system integration, most artificial structures can compete with their biological counterparts. However, the performance of artificial actuation still has significant gaps compared to the versatility of human actuators. To date, functionally high-performing humanoid-like robots rely on electric motors or hydraulic cylinders as these technologies were refined over the last century and are well available. However, due to the maturity of this technology, large leaps in performance are not expected. Further, functional geometric constraints of these technologies impede higher system integration with multifunctional structures.

The emerging soft actuation and sensing technology might comprise solutions to overcome these limitations. However, the technology requires more maturity to unfold the predicted potential and interfacing solutions between the components must be developed to be employed in complex robotic systems like humanoid robots (Rothemund et al., 2021).

Reliable, robust, and efficient robotic locomotion in rough and changing terrain experienced impressive progress during recent years substantially reducing the gap to the average human performance. In the authors’ opinion, robotic manipulation has a larger performance gap to cover. Robotic motor abilities to manipulate flexible structures (e.g., textiles) and to leverage the dexterity of more complex manipulators (e.g., shadow hand) are consistently improving. However, enhanced integration of tactile sensing and visual perception in manipulation control algorithms will be required to close the gap to the humans. Future research should invest in advancing the cognitive abilities of robots to understand the kinematic and semantic properties of environments allowing them to generate elaborate motion plans. Like humans, future robots might even be able to use and create tools that help them to improve motor, sensory and cognitive performance (Stilman et al., 2014).

## Conclusion

Despite the impressive advances during the last years, only some robots, which have been optimized for very specific tasks in semi-structured environments, reach performances that can compete with average humans in that specific task. However, no humanoid robot can compete with the dexterity and versatility of human movement functions. Such versatile functionality at the scale of humans might not be needed in certain industrial and rescue applications, where different specialized robotic systems collaborate or where environments are not human-centered and have been shaped or restricted to fit the robotic solutions. One could imagine that only minor restrictions and adaptations of human environments may let a humanoid robot perform faster and more accurately than humans. However, for the application of robots in natural (outdoor) environments, where humans usually stay, in environments that have been designed for humans or in applications where a robot closely cooperates with a human, a robotic system keeping up with the versatile nature of humans would have the best impact. Such applications could be elderly care in home settings, construction work in buildings, or any kind of household service.

To achieve such versatile functionality for locomotion and manipulation tasks performed by a robot at the scale of a human, a sufficiently tight packaging of the actuation and energy supply is required. Of course, environments could be structured in a way that robots can perform well, even with higher speed and accuracy, however, on the cost of versatility.

The major challenge will be the development of cognitive functions that understand the contexts and tasks in human-like environments based on onboard observations and that can derive meaningful action plans. This topic was not covered in this work.

In contrast, when looking into individual structures that are required to enable above-mentioned functions, the comparison sways to an advantage for robots–when ignoring the extra-ordinary regenerative and adaptive capabilities of human tissues. Sensing technology can outperform biological sensors in most aspects when combining different technologies. Joints have larger ranges of motion than human joints, and friction properties are comparable among both. Robotic links made from carbon or titanium outperform the stiffness and fracture properties of the human bone. Artificial actuators on their own are faster and have better endurance than the human muscle. However, the means for energy storage and power conversion required for the artificial actuators (e.g., reduction gearbox or compressor) prevent current actuation systems to achieve the required combination of speed, strength, and haptic accuracy at the weight, size, and efficiency of human muscles.

With respect to computational function, there is no single, stand-alone artificial computational system that can outperform the computational power of the human brain, especially when taking denominators mass, size, and power consumption into account, see Supplementary Appendix SB. However, robots might not need the full capacity required of human brains for the understanding of task-specific contexts, formulation of action plans, successful execution of sensory-motor functions, and dexterous movements.

Considering normalized values, then sensors, actuators, and skeletal systems perform excellently when comparing them with their biological counterparts. Consequently, one might ask, why the existing well-performing structures do not result in outperforming functions? The answer to this question can only be speculated at this time. Shaping the structural components to the scale of human system dimensions and integrating them in a compact design might be a limiting factor. One crucial disadvantage of robots could be the size and footprint of actuators including their power supplies and auxiliary components carried on the platform.

Conversely, one could conclude that at least tethered humanoids should have the potential to outperform any human, even the trained athlete, with respect to movement performance, dexterity, and versatility. However, it seems that even cabled humanoid robots are still far away from such a versatile performance level. Despite the remarkable progression of robotics over the last few years, scientists and engineers have not yet been successful in merging powerful structures and specialized solutions to obtain fully integrated dexterous and versatile systems that are comparable to the performance of humans. The persisting challenges involve processing vast sensory information into effective actuator commands; packaging and wiring large numbers of sensors and actuators into one compact system; implementing smart control strategies required to handle dexterous and versatile movement tasks. Another challenge is the dissipation of energy in computational units, actuators, and other moving parts that generate heat and need to be removed from the robotic device.

Versatile and dexterous robots that can outperform humans do not yet exist–they are still not among us. Hence, human skills prevail over robotic skills. New sensor fusion, machine learning, and decision-making strategies may be required to solve the current functional limitations of robots. However, it will still take some time for robots to assume universal skills, including human labor functions. This is especially true for service-oriented tasks within human-centered domains and in interactions with humans which demand versatile dexterity and a higher level of problem-solving cognitive performance.

## References

[B1] Ackerman E. (2015). ATLAS DRC robot is 75 percent new, completely unplugged. IEEE Spectrum. Available: https://spectrum.ieee.org/atlas-drc-robot-is-75-percent-new-completely-unplugged (Accessed February, 2022).

[B2] Ackerman E. Guizzo E. (2016). IEEE spectrum - schaft unveils awesome new bipedal robot at Japan conference. Available: https://spectrum.ieee.org/shaft-demos-new-bipedal-robot-in-japan.

[B3] Ackerman E. Guizzo E. (2022). IEEE spectrum - what robotics experts think of tesla’s Optimus robot. IEEE. Available: https://spectrum.ieee.org/robotics-experts-tesla-bot-optimus ([Accessed 2023].

[B4] Ackerman E. Guizzo E. (2021). New walk-man robot is slimmer, quicker, better at quenching your flames. Available: https://spectrum.ieee.org/new-version-of-walkman-is-slimmer-quicker-better-at-quenching-your-flames (Accessed February 22, 2022).

[B5] Acome E. Mitchell S. K. Morrissey T. Emmett M. Benjamin C. King M. (2018). Hydraulically amplified self-healing electrostatic actuators with muscle-like performance. Science 359 (6371), 61–65. 10.1126/science.aao6139 29302008

[B6] Aldebaran Documentation (2020). Nao – construction – aldebaran 2.1.4.13 documentation. Available: http://doc.aldebaran.com/2-1/family/robots/dimensions_robot (Accessed February 22, 2022).

[B7] Aldebaran (2008). NAO - battery. Available: http://doc.aldebaran.com/2-1/family/robots/battery_robot (Accessed February 22, 2022).

[B8] Ascento (2022). ASCENTO. Available: https://www.ascento.ch/([Accessed February].

[B9] ASIMO (2022). Specifications. ASIMO by Honda | the world's most advanced humanoid robot. Available: https://asimo.honda.com/asimo-specs/(Accessed February 22, 2022).

[B10] Aubin C. A. Gorissen B. Milana E. Buskohl P. R. Lazarus N. Slipher G. A. (2022). Towards enduring autonomous robots via embodied energy. Nature 602 (7897), 393–402. 10.1038/s41586-021-04138-2 35173338

[B11] Azibil Corporation (2023). Azibil - precision sensors for real-time position measurement with 0.1 μm accuracy. Azibil Corporation. Available: https://www.azbil.com/corporate/technology/techne/techne16_j1.html (Accessed August 13, 2023).

[B12] Barasuol V. Villarreal-Magana O. A. Sangiah D. Frigerio M. Baker M. Morgan R. (2018). Highly-integrated hydraulic smart actuators and smart manifolds for high-bandwidth force control. Front. Robotics AI 5, 51. 10.3389/frobt.2018.00051 PMC7919780 33659276

[B13] Barreira T. V. Rowe D. A. Kang M. others (2010). Parameters of walking and jogging in healthy young adults. Int. J. Exerc. Sci. 3 (1), 2.

[B14] Beckman E. M. Connick M. J. McNamee M. J. Parnell R. Tweedy S. M. (2017). Should Markus Rehm be permitted to compete in the long jump at the Olympic Games? Br. J. Sports Med. 51 (14), 1048–1049. 10.1136/bjsports-2016-096621 27474389

[B15] Beira R. Lopes M. Praça M. Santos-Victor J. Bernardino A. Metta G. (2006). “Design of the robot-cub (icub) head,” in Proceedings 2006 IEEE international conference on robotics and automation (ICRA 2006), 94–100.

[B16] Bekerman I. Gottlieb P. Vaiman M. (2014). Variations in eyeball diameters of the healthy adults. J. Ophthalmol. 2014, 1–5. 10.1155/2014/503645 PMC4238270 25431659

[B17] Billard A. Kragic D. (2019). Trends and challenges in robot manipulation. Science 364 (6446), eaat8414. 10.1126/science.aat8414 31221831

[B18] Biped humanoid robot group (2013). WABIAN-2R (2006-). Available: http://www.takanishi.mech.waseda.ac.jp/top/research/wabian/ (Accessed February 22, 2022).

[B19] Bjelonic M. Bellicoso C. D. de Viragh Y. Sako D. Tresoldi F. D. Jenelten F. (2019). Keep rollin’—whole-body motion control and planning for wheeled quadrupedal robots. IEEE Robotics Automation Lett. 4 (2), 2116–2123. 10.1109/lra.2019.2899750

[B20] Bjelonic M. Grandia R. Harley O. Galliard C. Zimmermann S. Hutter M. (2020). “Whole-body mpc and online gait sequence generation for wheeled-legged robots,” in 2021 IEEE/RSJ international conference on intelligent robots and systems (IROS).

[B21] Boston Dynamics (2022). Atlas™. Available: https://www.bostondynamics.com/atlas (Accessed February 22, 2022).

[B22] Boston Dynamics Support Center (2021). Spot specifications. Available: https://support.bostondynamics.com/s/article/Robot-specifications (Accessed February 22, 2022).

[B23] Boston Dynamics (2017). Youtube: introducing handle. Available: https://youtu.be/-7xvqQeoA8c (Accessed February, 2022).

[B24] BotJunkie (2008). Youtube: flame walking robot. Available: https://www.youtube.com/watch?v=LuwYkqHjioM (Accessed February 22, 2022).

[B25] Burt C. M. Piao X. Gaudi F. Busch B. Taufik N. (2008). Electric motor efficiency under variable frequencies and loads. J. irrigation drainage Eng. 134 (2), 129–136. 10.1061/(asce)0733-9437(2008)134:2(129)

[B26] Castillo G. A. Weng B. Zhang W. Hereid A. (2021). “Robust feedback motion policy design using reinforcement learning on a 3D Digit bipedal robot,” in IEEE/RSJ international conference on intelligent robots and systems (IROS).

[B27] Cameron J. R. Skofronick J. G. Grant R. M. Morin R. L. (2000). Physics of the body. Int. J. Med. Phys. Res. Pract. 27 (2), 425. 10.1118/1.598847

[B28] Cao M. Y. Laws S. Baena F. R. (2021). Six-Axis force/torque sensors for robotics applications: a review. IEEE Sensors J. 21 (24), 27238–27251. 10.1109/jsen.2021.3123638

[B29] Cao S. Zhis W. Wang X. (2009). Tensile properties of CFRP and hybrid FRP composites at elevated temperatures. J. Compos. Mater. 43 (4), 315–330. 10.1177/0021998308099224

[B30] Chen Y. Chen C. Rehman H. U. Zheng X. Li H. Liu H. (2020). Shape-memory polymeric artificial muscles: mechanisms, applications and challenges. Molecules 25 (18), 4246. 10.3390/molecules25184246 32947872 PMC7570610

[B31] Choi J.-H. Galea E. R. Hong W.-H. (2014). Individual stair ascent and descent walk speeds measured in a Korean high-rise building. Fire Technol. 50 (2), 267–295. 10.1007/s10694-013-0371-4

[B32] Choi J.-Y. Choi J.-P. Baek S.-H. (2009). Surgical accuracy of maxillary repositioning according to type of surgical movement in two-jaw surgery. Angle Orthod. 79 (2), 306–311. 10.2319/030608-136.1 19216601

[B33] Chou C.-P. Hannaford B. (1996). Measurement and modeling of McKibben pneumatic artificial muscles. IEEE Trans. robotics automation 12 (1), 90–102. 10.1109/70.481753

[B34] Chun S. Kim J.-S. Yoo Y. Choi Y. Jung S. J. Jang D. (2021). An artificial neural tactile sensing system. Nat. Electron. 4 (6), 429–438. 10.1038/s41928-021-00585-x

[B35] Collins S. Ruina A. Tedrake R. Wisse M. (2005). Efficient bipedal robots based on passive-dynamic walkers. Science 307 (5712), 1082–1085. 10.1126/science.1107799 15718465

[B36] Copper (2022). Copper development association inc. Available: https://www.copper.org/applications/industrial/bronze_bearing.html (Accessed February, 2022).

[B37] Coutant B. E. Westheimer G. (1993). Population distribution of stereoscopic ability. Ophthalmic Physiological Opt. 13 (1), 3–7. 10.1111/j.1475-1313.1993.tb00419.x 8510945

[B38] Cristiano J. Puig D. Garcia M. A. (2011). “On the maximum walking speed of NAO humanoid robots,” in XII workshop of physical agents, 3–7.

[B39] Cui J. Trinkle J. (2021). Toward next-generation learned robot manipulation. Sci. robotics 6 (54), eabd9461. 10.1126/scirobotics.abd9461 34043539

[B40] Curtin N. A. Bartlam-Brooks H. L. Hubel T. Y. Lowe J. C. Gardner-Medwin A. R. Bennitt E. (2018). Remarkable muscles, remarkable locomotion in desert-dwelling wildebeest. Nature 563 (7731), 393–396. 10.1038/s41586-018-0602-4 30356212

[B41] CYBATHLON (2022). Cybathlon. Available: Webpage https://cybathlon.ethz.ch/en (Accessed February, 2022).

[B42] Dahiya R. S. Metta G. Valle M. Sandini G. (2009). Tactile sensing—from humans to humanoids. IEEE Trans. robotics 26 (1), 1–20. 10.1109/tro.2009.2033627

[B43] Dargahi J. Najarian S. (2004). Human tactile perception as a standard for artificial tactile sensing—a review. Int. J. Med. robotics Comput. assisted Surg. 1 (1), 23–35. 10.1002/rcs.3 17520594

[B44] Das Gupta S. Bobbert M. F. Kistemaker D. A. (2019). The metabolic cost of walking in healthy young and older adults–A systematic review and meta analysis. Sci. Rep. 9 (1), 9956–10010. 10.1038/s41598-019-45602-4 31292471 PMC6620279

[B45] DLR 2022, "Toro," Available: https://www.dlr.de/rm/en/desktopdefault.aspx/tabid-11678/#gallery/28603. [Accessed 22 February 2022].

[B46] Durini D. Arutinov D. (2020). “Fundamental principles of photosensing,” in High performance silicon imaging, 3–24.

[B47] Dynamic Robotics Laboratory (2015). Youtube: ATRIAS robot: battery drain. Available: https://www.youtube.com/watch?v=se0nSJMZKcY (Accessed February 22, 2022).

[B48] Dynamics Laboratories (2015). Youtube: ATRIAS: an agile and efficient bipedal robot. Available: https://www.youtube.com/watch?v=YFEJvb8iM7A&t=70s (Accessed February 22, 2022).

[B49] Ellis K. J. (1990). Reference man and woman more fully characterized. Variations on the basis of body size, age, sex, and race. Nucl. Anal. Methods Life Sci. 26-27, 385–400. 10.1007/BF02992693 1704742

[B50] Engineering ToolBox (2022a). Friction - friction coefficients and calculator. Available: https://www.engineeringtoolbox.com/friction-coefficients-d_778.html#google_vignette (Accessed February 22, 2022).

[B51] Engineering Toolbox (2022b). Illuminance - recommended light level. Available: www.engineeringtoolbox.com/light-level-rooms-d_708.html (Accessed February, 2022).

[B52] Englsberger J. Werner A. Ott C. Henze B. Roa M. A. Garofalo G. (2014). “Overview of the torque-controlled humanoid robot TORO,” in 2014 IEEE-RAS international conference on humanoid robots, 916–923.

[B53] Zürich E. T. H., "Ascento," 2022. Available: https://www.ascento.ethz.ch/. [Accessed February 2022].

[B54] EurekAlert (2018). The humanoid robot walk-man for supporting emergency response teams. Available: https://www.eurekalert.org/news-releases/798389 (Accessed February 22, 2022).

[B55] Fakultät für Elektrotechnik und Informationstechnik (2022). ICUB. Available: https://www.ei.tum.de/ics/research/platforms/icub/ (Accessed February 22, 2022).

[B56] Ferwerda J. A. (2001). Elements of early vision for computer graphics. IEEE Comput. Graph. Appl. 21 (5), 22–33. 10.1109/38.946628

[B57] Gallagher S. Pollard J. Porter W. L. (2011). Locomotion in restricted space: kinematic and electromyographic analysis of stoopwalking and crawling. Gait posture 33 (1), 71–76. 10.1016/j.gaitpost.2010.09.027 20971644

[B58] Gallego G. Lund J. E. A. Mueggler E. Rebecq H. Delbruck T. Scaramuzza D. (2017). Event-based, 6-DOF camera tracking from photometric depth maps. IEEE Trans. pattern analysis Mach. Intell. 40 (10), 2402–2412. 10.1109/tpami.2017.2769655 29990121

[B59] Garrison W. M. (1990). Ultrahigh-strength steels for aerospace applications. Jom 42 (5), 20–24. 10.1007/bf03220942

[B60] Gawel A. Blum H. Pankert J. Kraemer K. Bartolomei L. Ercan S. (2019). “A fully-integrated sensing and control system for high-accuracy mobile robotic building construction,” in 2019 IEEE/RSJ international conference on intelligent robots and systems (IROS).

[B61] Gerndt R. Seifert D. Baltes J. H. Sadeghnejad S. Behnke S. (2015). Humanoid robots in soccer: robots versus humans in RoboCup 2050. IEEE Robotics Automation Mag. 22 (3), 147–154. 10.1109/mra.2015.2448811

[B62] Giancola S. Valenti M. Sala R. (2018). A survey on 3D cameras: metrological comparison of time-of-flight, structured-light and active stereoscopy technologies. Springer.

[B63] Gong Y. Hartley R. Da X. Hereid A. Harib O. Huang J.-K. (2019). “Feedback control of a cassie bipedal robot: walking, standing, and riding a segway,” in 2019 American control conference (ACC) (IEEE), 4559–4566.

[B64] Goswami A. Vadakkepat P. (2019). Humanoid robotics: a reference. Springer.

[B65] HABA (2021). Haba. Available: https://www.haba.ch/fileadmin/user_upload/Produktblaetter_deu/Alu_7075_DE.pdf (Accessed February, 2022).

[B66] Halodi Robotics (2021). “Youtube,” in Halodi robotics. Available at: https://youtu.be/FiSGOxDj1Sk ([Accessed 2023].

[B67] Ham R. V. Sugar T. G. Vanderborght B. Hollander K. W. Lefeber D. (2009). Compliant actuator designs. IEEE Robotics Automation Mag. 16 (3), 81–94. 10.1109/mra.2009.933629

[B68] Hassani H. Ghodsi M. Shadi M. Noroozi S. Dyer B. (2015). An overview of the running performance of athletes with lower-limb amputation at the Paralympic Games 2004--2012. Sports 3 (2), 103–115. 10.3390/sports3020103

[B69] Hemker T. Stelzer M. von Stryk O. Sakamoto H. (2009). Efficient walking speed optimization of a humanoid robot. Int. J. Robotics Res. 28 (2), 303–314. 10.1177/0278364908095171

[B70] Higueras-Ruiz D. R. Nishikawa K. Feigenbaum H. Shafer M. (2021). What is an artificial muscle? A comparison of soft actuators to biological muscles. Bioinspiration biomimetics 17 (1), 011001. 10.1088/1748-3190/ac3adf 34792040

[B71] Hill A. V. (1922). The maximum work and mechanical efficiency of human muscles, and their most economical speed. J. physiology 56 (1-2), 19–41. 10.1113/jphysiol.1922.sp001989 PMC1405410 16993552

[B72] Hinman M. R. O'Connell J. K. Dorr M. Hardin R. Tumlinson A. B. Varner B. (2014). Functional predictors of stair-climbing speed in older adults. J. Geriatric Phys. Ther. 37 (1), 1–6. 10.1519/jpt.0b013e318298969f 23835772

[B73] Hirschl R. B. (2015). The making of a surgeon: 10,000 hours? J. Pediatr. Surg. 50 (5), 699–706. 10.1016/j.jpedsurg.2015.02.061 25799086

[B74] Hobbelen D. De Boer T. Wisse M. (2008). “System overview of bipedal robots flame and tulip: tailor-made for limit cycle walking,” in 2008 IEEE/RSJ international conference on intelligent robots and systems (IEEE), 2486–2491.

[B75] Hogrel J.-Y. Payan C. A. Ollivier G. Tanant V. Attarian S. Couillandre A. (2007). Development of a French isometric strength normative database for adults using quantitative muscle testing. Archives Phys. Med. rehabilitation 88 (10), 1289–1297. 10.1016/j.apmr.2007.07.011 17908571

[B76] Hubicki C. Grimes J. Jones M. Renjewski D. Spröwitz A. Abate A. (2016). Atrias: design and validation of a tether-free 3d-capable spring-mass bipedal robot. Int. J. Robotics Res. 35 (12), 1497–1521. 10.1177/0278364916648388

[B77] Hunter I. Lafontaine S. Hollerbach J. Hunter P. (1991b). “Fast reversible NiTi fibers for use in microrobotics,” in Proc. 1991 IEEE micro electro mechanical systems-MEMS, 166–170.

[B78] Hunter I. W. Hollerbach J. M. Ballantyne J. (1991a). A comparative analysis of actuator technologies for robotics. Robot. Rev. 2, 299–342.

[B79] Hunter I. W. Lafontaine S. (1992). “A comparison of muscle with artificial actuators,” in Technical Digest IEEE solid-state sensor and actuator workshop (IEEE), 178–185.

[B80] Hutter M. Gehring C. Jud D. Lauber A. Bellicoso C. D. Tsounis V. (2016b). Anymal-a highly mobile and dynamic quadrupedal robot, 38–44.

[B81] Hutter M. Gehring C. Jud D. Lauber A. Bellicoso C. D. Tsounis V. (2016a). “Anymal-a highly mobile and dynamic quadrupedal robot,” in IEEE/RSJ international conference on intelligent robots and systems (IROS) (IEEE), 38–44.

[B82] Huxley A. (1980). Reflections on muscle. Liverpool University Press.

[B83] Hwangbo J. Lee J. Dosovitskiy A. Bellicoso D. Tsounis V. Koltun V. (2019). Learning agile and dynamic motor skills for legged robots. Sci. Robotics 4 (26), eaau5872. 10.1126/scirobotics.aau5872 33137755

[B84] IEEE Spectrum (2010). Hubo II humanoid robot is lighter and faster, makes his CREATOR PROUD. Available: https://spectrum.ieee.org/033010-hubo-ii-humanoid-robot-is-lighter-and-faster (Accessed February 22, 2022).

[B85] IEEE Spectrum ROBOTS (2022a). Cassie. Available: https://robots.ieee.org/robots/cassie (Accessed February 22, 2022).

[B86] IEEE Spectrum Robots (2021). Handle. Available: https://robots.ieee.org/robots/handle/(Accessed February, 2022).

[B87] IEEE Spectrum Robots (2022b). Lola. Available: https://robots.ieee.org/robots/lola (Accessed February 22, 2022).

[B88] IEEE Spectrum Robots (2018). Qrio. Available: https://robots.ieee.org/robots/qrio/ (Accessed February 22, 2022).

[B89] IEEE Spectrum Robots (2019). Spot. Available: https://robots.ieee.org/robots/spotmini/ (Accessed February 22, 2022).

[B90] i. HumanoidRobot (2010). Youtube. Available: https://youtu.be/JRqdIFCIZd8 (Accessed November 29, 2022).

[B91] ikinamo (2009a). Youtube - JO-ZERO robot kit: digInfo. Available: https://youtu.be/HDIkQ4o335Y ([Accessed 2023].

[B92] ikinamo (2009b). Youtube: JO-ZERO. Available: https://youtu.be/HDIkQ4o335Y (Accessed February 22, 2022).

[B93] Insta360 (2022). Insta360 ONE X2. Available at: https://www.insta360.com/de/product/insta360-onex2 (Accessed March, 2022).

[B94] Instituto Italiano di Technologia (2015). Youtube: IIT robot WALKMAN ready for the DARPA robotics challenge, 13. Available: https://www.youtube.com/watch?v=kZzwVwzAWME (Accessed February 22, 2022).

[B95] Instituto Italiano di Technologia (2018). Youtube: WALK-MAN operating in a damaged building. Available: https://www.youtube.com/watch?v=7UectbjusCI (Accessed February 22, 2022).

[B96] Intel Realsense (2021). Intel Realsense D415. Available: https://www.intelrealsense.com/depth-camera-d415/(Accessed February, 2022).

[B97] intuitive (2022). Da vinci surgical systems. Available: https://www.intuitive.com/en-us/products-and-services/da-vinci/systems (Accessed February 22, 2022).

[B98] Johnson M. Shrewsbury B. Bertrand S. Wu T. Duran D. Floyd M. (2015). Team IHMC's lessons learned from the DARPA robotics challenge trials. J. Field Robotics 32 (2), 192–208. 10.1002/rob.21571

[B99] Josephson R. K. (1985). Mechanical power output from striated muscle during cyclic contraction. J. Exp. Biol. 114 (1), 493–512. 10.1242/jeb.114.1.493

[B100] Kaneko K. Kanehiro F. Morisawa M. Akachi K. Miyamori G. Hayashi A. (2011). “Humanoid robot hrp-4-humanoid robotics platform with lightweight and slim body,” in 2011 IEEE/RSJ international conference on intelligent robots and systems, 4400–4407.

[B101] Kappassov Z. Corrales J.-A. Perdereau V. (2015). Tactile sensing in dexterous robot hands — review. Robotics Aut. Syst. 74, 195–220. 10.1016/j.robot.2015.07.015

[B102] Kashiri N. Abate A. Abram S. J. Albu-Schaffer A. Clary P. J. Daley M. (2018). An overview on principles for energy efficient robot locomotion. Front. Robotics AI 5, 129. 10.3389/frobt.2018.00129 PMC7805619 33501007

[B103] Katayon RADKHAH Maufroy C. Maus M. Scholz D. Seyfarth A. Von Stryk O. (2011). Concept and design of the Biobiped1 robot for human-like walking and running. Int. J. Humanoid Robotics 8 (3), 439–458. 10.1142/s0219843611002587

[B104] Kaufman J. (2013). The first 20 hours: how to learn anything fast!. Penguin.

[B105] Kawasaki H. Mouri T. (2019). Humanoid robot hand and its applied research. J. Robotics Mechatronics 31 (1), 16–26. 10.20965/jrm.2019.p0016

[B106] Kawasaki (2023). Kawasaki robotics YF003n. Available: https://kawasakirobotics.com/de/products-robots/yf003n/(Accessed May, 2023).

[B107] Kelley E. F. Lindfors M. Penczek J. (2006). Display daylight ambient contrast measurement methods and daylight readability. J. Soc. Inf. Disp. 14 (11), 1019–1030. 10.1889/1.2393026

[B108] Kim K. J. Shahinpoor M. (2003). Ionic polymer--metal composites: II. Manufacturing techniques. Smart Mater. Struct. 12 (1), 65–79. 10.1088/0964-1726/12/1/308

[B109] Kitano S. Hirose S. Horigome A. Endo G. (2016). TITAN-XIII: sprawling-type quadruped robot with ability of fast and energy-efficient walking, 3. Springer, 1–16.

[B110] Koyo Bearings/JTEKT CORPORATION (2022). Frictional coefficient (reference): basic bearing knowledge: koyo Bearings. Available: https://koyo.jtekt.co.jp/en/support/bearing-knowledge/8-4000.html (Accessed February 22, 2022).

[B111] Kwartowitz D. M. Herrell S. D. Galloway R. L. (2007). Update: toward image-guided robotic surgery: determining the intrinsic accuracy of the daVinci-S robot. Int. J. Comput. Assisted Radiology Surg. 1 (5), 301–304. 10.1007/s11548-006-0064-2

[B112] Kwon W. Kim H. K. Park J. K. Roh C. H. Lee J. Park J. (2007). “Biped humanoid robot Mahru III,” in 2007 7th IEEE-RAS international conference on humanoid robots, 583–588.

[B113] Leah T. Emily M. Kenton R. (2017). Static and dynamic validation of inertial measurement units. Gait Posture 57, 80–84. 10.1016/j.gaitpost.2017.05.026 28578138

[B114] Leng X. Hu X. Zhao W. An B. Zhou X. Liu Z. (2021). Recent advances in twisted-fiber artificial muscles. Adv. Intell. Syst. 3 (5), 2000185. 10.1002/aisy.202000185

[B115] Li G. Liu S. Wang L. Zhu R. (2020b). Skin-inspired quadruple tactile sensors integrated on a robot hand enable object recognition. Sci. Robotics 5 (49), eabc8134. 10.1126/scirobotics.abc8134 33328298

[B116] Li Q. Kroemer O. Su Z. Veiga F. F. Kaboli M. Ritter H. J. (2020a). A review of tactile information: perception and action through touch. IEEE Trans. Robotics 36, 1619–1634. 10.1109/tro.2020.3003230

[B117] Li Z.-M. (2006). Functional degrees of freedom. Mot. control 10 (4), 301–310. 10.1123/mcj.10.4.301 17293614

[B118] Liang W. Liu H. Wang K. Qian Z. Ren L. Ren L. (2020). Comparative study of robotic artificial actuators and biological muscle. Adv. Mech. Eng. 12 (6), 168781402093340. 10.1177/1687814020933409

[B119] Lundeberg S. (2021). Oregon state university. Available: https://today.oregonstate.edu/news/bipedal-robot-developed-oregon-state-makes-history-learning-run-completing-5kSteve (Accessed March, 2022).

[B120] Luo R. Wang C. Schwarm E. Colin K. Evelyn M. Kaveti P. (2022). “Towards robot avatars: systems and methods for teleinteraction at avatar XPRIZE semi-finals,” in 2022 IEEE/RSJ international conference on intelligent robots and systems (IROS) (IEEE), 7726–7733.

[B121] Luo S. Bimbo J. Dahiya R. Liu H. (2017). Robotic tactile perception of object properties: a review. Mechatronics 48 (54-67), 54–67. 10.1016/j.mechatronics.2017.11.002

[B122] Lupton H. (1922). The relation between the external work produced and the time occupied in a single muscular contraction in man. J. Physiology 57 (1-2), 68–75. 10.1113/jphysiol.1922.sp002043 PMC1405450 16993600

[B123] Madden J. D. (2007). Mobile robots: motor challenges and materials solutions. science 318 (5853), 1094–1097. 10.1126/science.1146351 18006737

[B124] Meghdari A. Alemi M. Zakipour M. Kashanian S. A. (2019). Design and realization of a sign language educational humanoid robot. J. Intelligent Robotic Syst. 95 (1), 3–17. 10.1007/s10846-018-0860-2

[B125] Michigan Robotics (2020). “Dynamic legged locomotion lab,” in Youtube: 2.1 m/s = walking speed achieved by bipedal robot Cassie blue. Available: https://youtu.be/lu_jcL84wNE (Accessed March, 2022).

[B126] Miki T. Lee J. Hwangbo J. Wellhausen L. Koltun V. Hutter M. (2022). Learning robust perceptive locomotion for quadrupedal robots in the wild. Sci. Robotics 7 (62), eabk2822. 10.1126/scirobotics.abk2822 35044798

[B127] MIT News | Massachusetts Institute of Technology (2013). MIT ‘cheetah’ ROBOT rivals running animals in efficiency. Available: https://news.mit.edu/2013/mit-cheetah-robot-0308 (Accessed February 22, 2022).

[B128] Morimoto J. Atkeson C. G. Endo G. Cheng G. (2007). “Improving humanoid locomotive performance with learnt approximated dynamics via Gaussian processes for regression,” in 2007 IEEE/RSJ international conference on intelligent robots and systems, 4234–4240.

[B129] Muscolo G. G. Recchiuto C. T. Hashimoto K. Dario P. Takanishi A. (2012). “Towards an improvement of the SABIAN humanoid robot: from design to optimization,” in Journal of mechanical engineering and automation (Scientific & Academic Publishing), 2, 80–84.

[B130] Nakashima M. Kuwahara K. (2016). Realization and swimming performance of the breaststroke by a swimming humanoid robot. Robomech J. 3 (1), 10. 10.1186/s40648-016-0049-z

[B131] NED Directory (2023). NED directory. Available: https://directory.newequipment.com/classified/digit-the-bipedal-humanoid-robot-256142.html ([Accessed 2023].

[B132] Nestik T. Zhuravlev A. Eduard P. Marianna S. C. Lioudmila B. Piurcosky F. P. (2018). Technophobia as a cultural and psychological phenomenon: theoretical analysis. Interação-Revista De. Ensino, Pesqui. E Extensão 20 (1), 266–281. 10.33836/interacao.v20i1.191

[B133] Ota Y. (2010). “Toyota partner robots — development and implementation vision,” in Mobile robotics: solutions and challenges, 7–8.

[B134] Ouster (2021). Ouster - OS2 sensor. Available: https://ouster.com/products/scanning-lidar/os2-sensor/(Accessed February, 2022).

[B135] PAL Robotics (2022). REEM-C – PAL robotics: leading service robotics. Available: https://pal-robotics.com/robots/reem-c/(Accessed February 22, 2022).

[B136] Phantom (2022). Phantom v2512. Available: https://www.phantomhighspeed.com/products/cameras/ultrahighspeed/v2512 (Accessed February 22, 2022).

[B137] Plastic Pals (2022). Youtube: SWUMANOID (swimming humanoid robot)," 1 june 2012. Available: https://www.youtube.com/watch?v=e_Gp5h14KG4 (Accessed February 22, 2022).

[B138] Playter R. Buehler M. Raibert M. (2006). “BigDog,” in Unmanned systems technology VIII (SPIE), 896–901.

[B139] Pontes J. P. D. C. (2019). A hybrid and adaptive approach to humanoid locomotion: blending rhythmic primitives and feet placement strategies.

[B140] Poppy Mosbacher (2020). Youtube: nao robot does Tai chi. Available: https://www.youtube.com/watch?v=t4L5z4r7QS8 (Accessed February 22, 2022).

[B141] Potter M. C. Wyble B. Hagmann C. E. McCourt E. S. (2014). Detecting meaning in RSVP at 13 ms per picture. Atten. Percept. Psychophys. 76 (2), 270–279. 10.3758/s13414-013-0605-z 24374558

[B142] Radhakrishnan V. (1998). Locomotion: dealing with friction. Proc. Natl. Acad. Sci. 95 (10), 5448–5455. 10.1073/pnas.95.10.5448 9576902 PMC20397

[B143] Raibert M. H. (1986). Legged robots that balance. MIT press.

[B144] Rakita D. Mutlu B. Gleicher M. Hiatt L. M. (2019). Shared control--based bimanual robot manipulation. Sci. Robotics 4 (30), eaaw0955. 10.1126/scirobotics.aaw0955 33137728

[B145] Reed K. L. Brown T. D. (2001). Elastic modulus and strength of emu cortical bone. Iowa Orthop. J. 21, 53–57. 11813952 PMC1888202

[B146] Riddick R. Kuo A. (2022). Mechanical work accounts for most of the energetic cost in human running. Sci. Rep. 12 (1), 645. 10.1038/s41598-021-04215-6 35022431 PMC8755824

[B147] Riener R. Harders M. (2012). “Introduction to virtual reality in medicine,” in Virtual reality in medicine, 1–12.

[B148] Riener R. (2016). The Cybathlon promotes the development of assistive technology for people with physical disabilities. J. neuroengineering rehabilitation 13 (1), 1–4. 10.1186/s12984-016-0157-2 PMC4886429 27246601

[B149] Robotis (2023). Robotis. Robotis, Available at: https://emanual.robotis.com/docs/en/platform/op2/getting_started/([Accessed 2023].

[B150] ROBOTIS (2022). ROBOTIS OP2. Available: https://www.robotis.com/model/page.php?co_id=prd_op2 (Accessed February 22, 2022).

[B151] Robots G. (2021). Rechargeable battery for the NAO PROGRAMMABLE humanoid robot. Available: https://www.generationrobots.com/en/401790-rechargeable-battery-for-the-nao-programmable-humanoid-robot (Accessed February 22, 2022).

[B152] Robots (2019). IEEE. Available: https://robotsguide.com/robots/digit ([Accessed 2023].

[B153] Romualdi G. Dafarra S. Hu Y. Pucci D. (2018). “A benchmarking of dcm based architectures for position and velocity controlled walking of humanoid robots,” in 2018 IEEE-RAS 18th international conference on humanoid robots (humanoids), 1–9.

[B154] Rosenthal E. Solomon R. J. Johnson C. (2004). Waves vs. Photons: a new view of how we detect light and color. United States: Creative Technologies LLC.

[B155] Rothemund P. Kim Y. Heisser R. H. Zhao X. Shepherd R. F. Keplinger C. (2021). Shaping the future of robotics through materials innovation. Nat. Mater. 20 (12), 1582–1587. 10.1038/s41563-021-01158-1 34815572

[B156] Rothling F. Haschke R. Steil J. J. Ritter H. (2007). “Platform portable anthropomorphic grasping with the bielefeld 20-DOF shadow and 9-DOF TUM hand,” in 2007 IEEE/RSJ international conference on intelligent robots and systems (IEEE), 2951–2956.

[B157] Sagbas B. (2016). Biotribology of artificial hip joints. InTechOpen.

[B158] Sakagami Y. Watanabe R. (1999). “The intelligent asimo: system overview and integration robots and system,” in IEEE/RSJ int. Conf. On intelligent robots and system (IEEE), 813–830.

[B159] Samson M. M. Crowe A. De Vreede P. L. Dessens J. A. G. Duursma S. A. Verhaar H. J. J. (2001a). Differences in gait parameters at a preferred walking speed in healthy subjects due to age, height and body weight. Aging Clin. Exp. Res. 13 (1), 16–21. 10.1007/bf03351489 11292147

[B160] Samson M. M. Crowe A. De Vreede P. L. Dessens J. A. G. Duursma S. A. Verhaar H. J. J. (2001b). Differences in gait parameters at a preferred walking speed in healthy subjects due to age, height and body weight. height body weight 13, 16–21. 10.1007/bf03351489 11292147

[B161] Scharnowski F. Hermens F. Herzog M. H. (2007). Bloch’s law and the dynamics of feature fusion. Vis. Res. 47 (18), 2444–2452. 10.1016/j.visres.2007.05.004 17675130

[B162] Schiller P. H. Slocum W. M. Jao B. Weiner V. S. (2011). The integration of disparity, shading and motion parallax cues for depth perception in humans and monkeys. Brain Res. 1377, 67–77. 10.1016/j.brainres.2011.01.003 21219887 PMC3047464

[B163] Schoenfeld B. J. Grgic J. Van Every D. W. Plotkin D. L. (2021). Loading recommendations for muscle strength, hypertrophy, and local endurance: a re-examination of the repetition continuum. Sports 9 (2), 32. 10.3390/sports9020032 33671664 PMC7927075

[B164] Seifert L. Leblanc H. Chollet D. Delignières D. (2010). Inter-limb coordination in swimming: effect of speed and skill level. Hum. Mov. Sci. 29 (1), 103–113. 10.1016/j.humov.2009.05.003 19945188

[B165] Seok S. Wang A. Chuah M. Y. Hyun D. J. Lee J. Otten D. M. (2014). Design principles for energy-efficient legged locomotion and implementation on the MIT cheetah robot. Ieee/asme Trans. mechatronics 3 (20), 1117–1129. 10.1109/tmech.2014.2339013

[B166] Serway R. A. Vuille C. (2014). College physics. Cengage Learning.

[B167] Seyfarth A. Iida F. Tausch R. Stelzer M. von Stryk O. Karguth A. (2009). Towards bipedal jogging as a natural result of optimizing walking speed for passively compliant three-segmented legs. Int. J. Robotics Res. 28 (2), 257–265. 10.1177/0278364908095843

[B168] Sharbafi M. A. Rode C. Kurowski S. Scholz D. Möckel R. Radkhah K. (2016). A new biarticular actuator design facilitates control of leg function in BioBiped3. Bioinspiration Biomimetics 11 (4), 046003. 10.1088/1748-3190/11/4/046003 27367459

[B169] Signe Brewster (2015). Youtube: NASA jet propulsion lab robosimian robot drives car at DARPA robotics challenge. Available: https://www.youtube.com/watch?v=PBsfJZ-Gixc (Accessed February 22, 2022).

[B170] Skorka O. Joseph D. (2011). Toward a digital camera to rival the human eye. J. Electron. Imaging 20 (3), 33009.

[B171] Sleiman J.-P. Carius J. Grandia R. Wermelinger M. Hutter M. (2019). “Contact-implicit trajectory optimization for dynamic object manipulation,” in 2019 IEEE/RSJ international conference on intelligent robots and systems (IROS) (IEEE), 6814–6821.

[B172] Smith J. Sharf I. Trentini M. (2006). “PAW: a hybrid wheeled-leg robot,” in Proceedings 2006 IEEE international conference on robotics and automation, 2006. ICRA 2006 (IEEE), 4043–4048.

[B173] Smith Marshal and Marshal Smith Technology (2018). Atlas and SpotMini more agile than EVER, running and climbing stairs. Available: https://www.industrytap.com/atlas-spotmini-agile-ever-running-climbing-stairs/45464 (Accessed February, 2022).

[B174] Snyder W. Cook M. Nasset E. Karhausen L. Tipton I. (1974). Report of the task group on reference man. Rep. Prep. Int. Comm. Radiological Prot. 23, 46–57.

[B175] Spillmann L. Werner J. S. (2012). Visual perception: the neurophysiological foundations. Elsevier.

[B176] Stasse O. Brousse E. Naveau M. Régnier R. Avrin G. (2018). Souères and others, "Benchmarking the hrp-2 humanoid robot during locomotion. Front. Robotics AI, 122. 10.3389/frobt.2018.00122 PMC7805704 33501001

[B177] Steudel-Numbers K. L. Wall-Scheffler C. M. (2009). Optimal running speed and the evolution of hominin hunting strategies. J. Hum. Evol. 56 (4), 355–360. 10.1016/j.jhevol.2008.11.002 19297009

[B178] Stilman M. Zafar M. Erdogan C. Hou P. Reynolds-Haertle S. Tracy G. (2014). “Robots using environment objects as tools the ‘MacGyver’ paradigm for mobile manipulation,” in 2014 IEEE international conference on robotics and automation (ICRA) (IEEE), 2568.

[B179] Swiss Alps 100 (2021). Swiss Alps 100. Available: https://www.swissalps100.com/results.asp?iY=2021&iD=160 (Accessed February, 2022).

[B180] Swiss Mile (2022). Swiss mile. Available: https://www.swiss-mile.com/ (Accessed February, 2022).

[B181] Swiss State Secretariat for Economic Affairs (2016). State Secretariat for economic Affairs. Available: https://www.seco.admin.ch/dam/seco/de/dokumente/Arbeit/Arbeitsbedingungen/Arbeitsgesetz%20und%20Verordnungen/Wegleitungen/Wegleitungen%203/ArGV3_art25.pdf.download.pdf/ArGV3_art25_de.pdf (Accessed February, 2022).

[B182] Syawaludin M. F. Kim C. Hwana J.-I. (2019). “Hybrid camera system for telepresence with foveated imaging,” in 2019 IEEE conference on virtual reality and 3D user interfaces (VR), 1173–1174.

[B183] Szkopek J. Redlarski G. (2019). Artificial-hand technology—current state of knowledge in designing and forecasting changes. Appl. Sci. 9 (19), 4090. 10.3390/app9194090

[B184] Takiyama K. Yokoyama H. Kaneko N. Nakazawa K. (2020). Speed-dependent and mode-dependent modulations of spatiotemporal modules in human locomotion extracted via tensor decomposition. Sci. Rep. 10 (1), 680. 10.1038/s41598-020-57513-w 31959831 PMC6971295

[B185] Tektronics Expert (2016). IIT, tektronix join forces to bring battery power to icub robot platform. Available: https://www.tek.com/blog/iit-tektronix-join-forces-bring-battery-power-icub-robot-platform (Accessed February 22, 2022).

[B186] The Verge (2015). Youtube: the 2015 DARPA robotics challenge finals. Available: https://youtu.be/8P9geWwi9e0 (Accessed February, 2022).

[B187] The world material (2022). The world material. Available: https://www.theworldmaterial.com/density-of-metals/ (Accessed February, 2022).

[B188] TheIHMC (2019). Youtube - jerry pratt: towards humanoid avatar robots for Co-exploration of hazardous environments. Available: https://www.youtube.com/watch?v=HefjKANiZx0&t=405s (Accessed August 13, 2023).

[B189] Thomson Reuters (2015). Walk-man the humanoid walks tall. Available: https://www.reuters.com/article/taly-walkman-robot-idUSKBN0TG1KB20151127 (Accessed February 22, 2022).

[B190] Tktronix (2022). IIT, tektronix join forces to bring battery power to iCub robot platform. Available: https://www.tek.com/blog/iit-tektronix-join-forces-bring-battery-power-icub-robot-platform (Accessed February 22, 2022).

[B191] Todd T. W. Beecher H. Williams G. H. Todd A. W. (1940). The weight and growth of the human eyeball. Hum. Biol. 12 (1), 1–20.

[B192] Toyota Motor (2022). Toyota announces overview of 'Toyota PARTNER ROBOT. Available: https://global.toyota/en/detail/245497 (Accessed February, 2022).

[B193] Tranzatto M. Mascarich F. Bernreiter L. Godinho C. Camurri M. Khattak S. (2022). Cerberus: autonomous legged and aerial robotic exploration in the tunnel and urban circuits of the darpa subterranean challenge. *arXiv preprint arXiv:2201.07067* .

[B194] TU DELFT (2022). Flame. Available: http://www.3me.tudelft.nl/live/pagina.jsp?id=01f7247a-ae48-4328-a17f-631c0e8c38af& lang=en (Accessed February 22, 2022).

[B195] Tucker V. A. (1975). The energetic cost of moving about: walking and running are extremely inefficient forms of locomotion. Much greater efficiency is achieved by birds, fish—and bicyclists. Am. Sci. 63 (4), 413–419. 1137237

[B196] Vallery H. Veneman J. Van Asseldonk E. Ekkelenkamp R. Buss M. Van Der Kooij H. (2008). Compliant actuation of rehabilitation robots. IEEE Robotics Automation Mag. 15 (3), 60–69. 10.1109/mra.2008.927689

[B197] Vanderborght B. Albu-Schaeffer A. Bicchi A. Burdet E. Caldwell D. Carloni R. (2013). Variable impedance actuators: a review. Robotics Aut. Syst. 61 (12), 1601–1614. 10.1016/j.robot.2013.06.009

[B198] Venture G. Nakamura Y. Yamane K. Hirashima M. (2007). “A painless and constraint-free method to estimate viscoelastic passive dynamics of limbs' joints to support diagnosis of neuromuscular diseases,” in 2007 29th annual international conference of the IEEE engineering in medicine and biology society (IEEE), 5362–5365. 10.1109/IEMBS.2007.4353553 18003219

[B199] Verstraten T. Beckerle P. Furnémont R. Mathijssen G. Vanderborght B. Lefeber D. (2016). Series and Parallel Elastic Actuation: impact of natural dynamics on power and energy consumption. Mech. Mach. Theory 102, 232–246. 10.1016/j.mechmachtheory.2016.04.004

[B200] Wevolver (2022). Durus. Available: https://www.wevolver.com/wevolver.staff/durus (Accessed February 22, 2022).

[B201] Wisth D. Camurri M. Fallon M. (2023). VILENS: visual, inertial, lidar, and leg odometry for all-terrain legged robots. IEEE Trans. Robotics 39 (1), 309–326. 10.1109/tro.2022.3193788

[B202] Wolf P. Riener R. (2018). Cybathlon: how to promote the development of assistive technologies. Sci. Robotics 3 (17), eaat7174. 10.1126/scirobotics.aat7174 33141744

[B203] World Athletics (2022). World athletics. Available: https://www.worldathletics.org/records/all-time-toplists/road-running/marathon/outdoor/men/senior (Accessed November 15, 2022).

[B204] World Health Organisation (2007). International classification of functioning, disability, and health: children & youth version: ICF-CY. World Health Organization.

[B205] World Health Organisation (2018). WHO housing and health guidelines. World Health Organization.

[B206] Xia Z. Deng Z. Fang B. Yang Y. Sun F. (2022). A review on sensory perception for dexterous robotic manipulation. Int. J. Adv. Robotic Syst. 19 (2), 172988062210959. 10.1177/17298806221095974

[B207] Xie Z. Clary P. Dao J. Morais P. Hurst J. van de Panne M. (2019). Iterative reinforcement learning based design of dynamic locomotion skills for cassie. *arXiv preprint arXiv:1903.09537* .

[B208] Yamane K. Fujita Y. Nakamura Y. (2005). “Estimation of physically and physiologically valid somatosensory information,” in Proceedings of the 2005 IEEE international conference on robotics and automation, 2624–2630.

[B209] Yamane K. Murai A. (2016). “A comparative study between humans and humanoid robots,” in Humanoid robotics: a reference (Dordrecht: Springer), 873–892.

[B210] Yan Y. Hu Z. Yang Z. Yuan W. Song C. Pan J. (2021). Soft magnetic skin for super-resolution tactile sensing with force self-decoupling. Sci. Robotics 6 (51), eabc8801. 10.1126/scirobotics.abc8801 34043530

[B211] Yin H. Varava A. Kragic D. (2021). Modeling, learning, perception, and control methods for deformable object manipulation. Sci. Robotics 6 (54), eabd8803. 10.1126/scirobotics.abd8803 34043538

[B212] Youn J.-H. Jeong S. M. Hwang G. Kim H. Hyeon K. Park J. (2020). Dielectric elastomer actuator for soft robotics applications and challenges. Appl. Sci. 10 (2), 640. 10.3390/app10020640

[B213] Youtube - Tesla Bot Update (2023). Tesla. Available at: https://youtu.be/XiQkeWOFwmk?si=W-nFkW652nTswalM ([Accessed 2023].

[B214] Yuan W. Li R. Srinivasan M. A. Adelson E. H. (2015). “Measurement of shear and slip with a GelSight tactile sensor,” in 2015 IEEE international conference on robotics and automation (ICRA) (IEEE), 304–311.

[B215] Zimmermann Y. Forino A. Riener R. Hutter M. (2019). ANYexo: a versatile and dynamic upper-limb rehabilitation robot. IEEE Robotics Automation Lett. 4 (4), 3649–3656. 10.1109/lra.2019.2926958

