# Supplementary material for: Do robots outperform humans in human-centered domains?
Source: Front Robot AI. 2023 Nov 7;10:1223946. doi: 10.3389/frobt.2023.1223946 (PMC10661952; doi:10.3389/frobt.2023.1223946)
Supplement: Supplementary file 1 [file DataSheet1.PDF]

APPENDIX A: Estimation of Stair Climbing Speeds

The speed of climbing stairs was mostly not specifically measured or published by the robot manufacturers. Therefore, we estimated these values for 7 of the collected robots that are capable of climbing stairs based on the observation of existing video material. In most of those sources, step sizes corresponded to those of human environments, i.e., 15 – 20 cm [1], except with Atlas and NAO:

1. Asimo: A YouTube video [2] shows Asimo during a performance show climbing 5 steps of a staircase used in human indoor environments in about 5 seconds, thus, achieving a stair climbing speed of 1 steps/s.

2. HRP Robot: A YouTube video [3] shows HRP-4 climbing 5 steps of a staircase reproduced from an industrial setup in a laboratory environment. The step heights are 18.5 cm (only the last step is 14.5 cm), which correspond to the typical step heights in human environments. It takes the robot about 19 seconds to climb the 5 steps, thus, achieving a stair climbing speed of 0.26 steps/s.

3. HUBO: A YouTube video [4] shows DRC-HUBO climbing 4 steps of an industrial staircase as used by humans within the contest of the DARPA Robotics Challenge in about 120 seconds, thus, achieving a stair climbing speed of 0.03 steps/s.

4. NAO: A YouTube video [5] shows NAO, a doll-sized humanoid, climbing 5 steps of a properly sized staircase (scaled to fit the size of the robot, with no detailed information availale) in about 4 minutes and 24 seconds, thus, achieving a stair climbing speed of 0.02 steps/s.

5. Atlas: A YouTube video [6] shows Atlas performing highly athletic maneuvers, including climbing 3 high steps (estimated to be around 40 cm in step height) in about 3 seconds, thus, achieving a stair climbing speed of approximately 1 steps/s.

6. Cassie: A YouTube video [7] shows Cassie robot climbing a laboratory set-up staircase, with19 cm high steps. The robots climbs 3 steps in about 2 seconds, thus, achieving a stair climbing speed of 1.5 steps/s.

APPENDIX B: Non-haptic interaction and conversation

Results: Function

Applying Knowledge

When focusing on a single task, humans can maintain attention for the duration of up to 20 minutes [8] and every attention shift to a different task takes between 50 ms and 1600 ms- [9]. Human multitasking negatively affects performance [10], and most humans cannot switch very quickly between different single tasks. Machines can outperform humans both in task endurance and in multitasking functions that require several independently running computational processes.

Reading and writing are trivial tasks for machines, once the information is available in a well-defined structure, while trained humans are limited to read around 330 words per minute (wpm), handwrite 20 wpm, and type up to 80 wpm [11]. However, humans are still more capable in perceiving and processing unstructured information, e.g., to extract semantic information from a visual observation (identification of an objects function). However, humans are limited to a low number of operations per second (op/s), while machines can outperform the human easily, being able to perform billions of operations per second (op/s).

Conversation

Thanks to wired and wireless technologies exceeding 1’000 Gbit/s, machine-machine communication is virtually immediate, so that information can be transferred easily and rapidly. Machines communicate also with humans and vice versa in the form of human-like interactions. Human spoken language transfers about 39 bit/s on average [12], and it is estimated that only about 7% of whole human communication takes place in the verbal channel [13]. Regarding robotic verbal communication, improvements have been made thanks to deep learning approaches in natural language processing, but the “grounding problem” of language semantics which connects words to their meanings is still limiting the performance of chatbots and virtual assistants [14]. Whereas robots and machines such as smart phones can “understand” spoken language, they are far from understanding human non-verbal signs as transported via gestures and mimics.

Results: Structures

Computational System

The human brain is a powerful and barely understood organ that is capable of computing and storing information with high performance (estimates of 10^{28} operations per second and 8^{19} bits of memory) [15], comparatively small volume (1.2 l) [16], small mass (1.3 kg) [17], and with very little power consumption (20 W) [18]. It is difficult to compare the human brain with artificial computational systems because they work on completely different substrates (3D biological cell structures *vs* 2D silicon structures) and with completely different paradigms. In this paper, we compare biological with artificial “brains” using the features of computational power expressed by the number of operations per second (FLOPS) and memory storage expressed in bits.

There is no single, stand-alone artificial computational system that can outperform the computational power of the brain (Fig. 5). Smartphones are smaller and more energy efficient than the human brain, but neither their computational power nor their memory storage capacity can compete with the human brain. With respect to computational memory, only the supercomputer *SUMMIT* can reach performance levels comparable

to that of a human brain, however, at a cost of an extremely large size, weight, and power consumption preventing its use on a mobile humanoid platform [19].

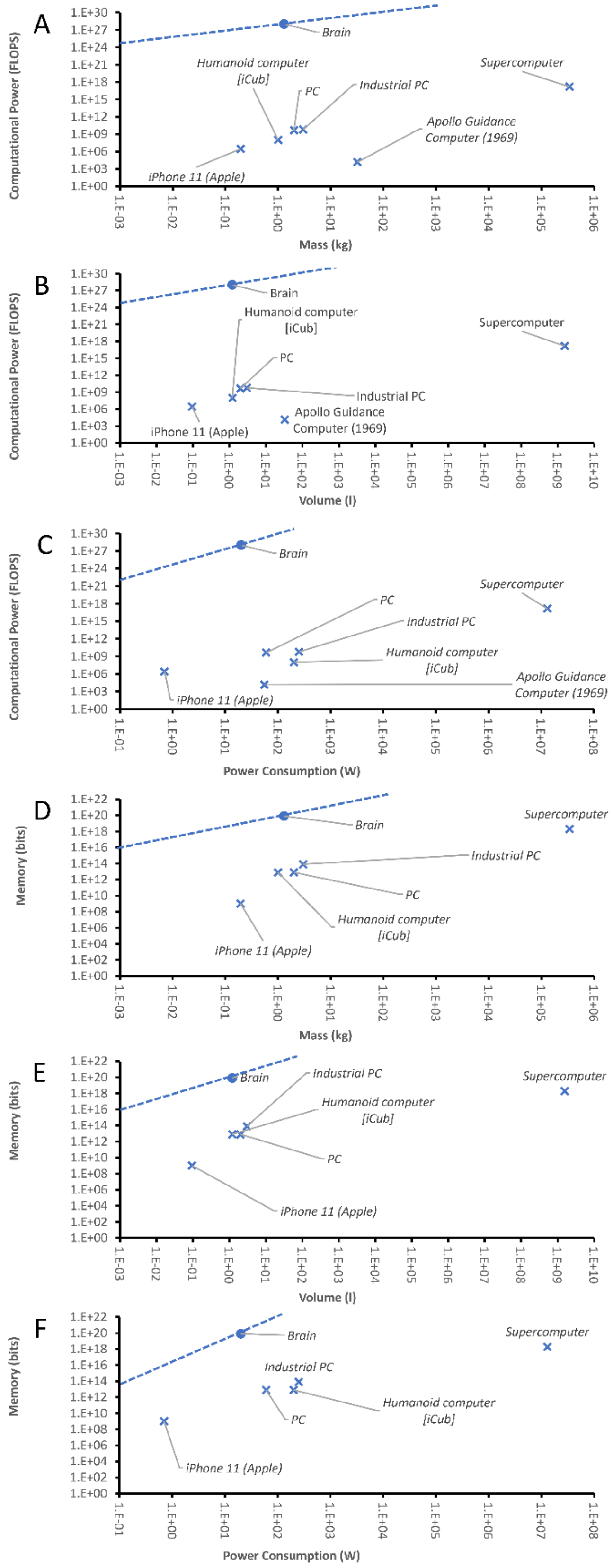

Fig. 4. Computational performance decomposed into the features computational power measured in FLOPS (number of operations per second) and memory storage capacity measured

in bits. Features are normalized by denominators mass, size (i.e., volume) and power consumption. In the case of the smartphone, PC, and industrial PC, estimated sample values have been chosen to represent the performance of current technologies. Computational power and memory of the brain are estimates, chosen from the most recent sources in the literature [20] [21] [22] [23] [24]. Computational power and memory of the human brain are taken from [25] [15].

Hearing Systems

Hearing systems can be evaluated with respect to the features frequency range, detection threshold, or localization accuracy of sound source. For the human hearing system [26] the detection threshold is 20 mPa (i.e., 0 Db SPL – sound pressure level) and the detectable frequency range spans from to 20 Hz – 20 kHz. Sound source localization in the horizontal plane has an accuracy of about 1° in the frontal area of the head and around 15° degrees on the side.

All these features can be matched by current microphones. Sound source localization can be achieved with an accuracy between 1.6° to 30° and depends upon the number and configuration of microphones [27] [28]. However, high accuracy sound source detection methods require larger spatial constructions. When artificial systems are used in humanoid robots, with microphones placed at distances that are comparable to the distance of the human ears, the performance of sound source localization is expected to be like that of humans [29].

Sound Generating Systems

Sound producing structures exist mostly for communication purposes. In humans, the organ of speech is constituted by the vocal cords. Air flow, produced by muscular activities at the lungs and diaphragm, brings the cords to vibrate so that sound is produced. During speech sound frequencies range from 85 Hz to several thousands of Hz. Frequency contents up to 22 kHz can be relevant in human speech [30]. Typical fundamental frequencies (first mode) of human speech reach from 128 Hz to 267 Hz [31], with an average sound pressure intensity of 60 dB [32] [33]. Artificial sound generating systems can cover broader ranges and larger intensities at lower weight, size, and power consumption than the human organ of speech.

References

[1] SUVA, „Sichere Treppen für sichere Betriebe,“ 2022. [Online]. Available: https://www.suva.ch/de-CH/material/Factsheets/sichere-treppen-fuer-sichere-betriebe. [Zugriff am March 2022].

[2] Steve Diggins, „ASIMO robot walking up and down stairs,“ 28 June 2014. [Online]. Available: https://www.youtube.com/watch?v=ixsliyqpYYY. [Zugriff am 22 February 2022].

[3] IDH LIRMM, „Youtube: Humanoid Robot Hrp-4 Climbs 18.5 CM Stairs,“ 25 October 2018. [Online]. Available: https://www.youtube.com/watch?v=vFCFKAunsYM. [Zugriff am 22 February 2022].

[4] IEEE Spectrum, „Youtube: Team KAIST’s DRC-HUBO Robot Completes Stairs Task,“ 3 June 2019. [Online]. Available: https://www.youtube.com/watch?v=UPxZO8NA6o0. [Zugriff am 22 February 2022].

[5] HumanoidsFreiburg, „Youtube: NAO Humanoid Climbing a Full Spiral STAIRCASE Autonomously," 23 July 2012. [Online]. Available: https://www.youtube.com/watch?v=U9ll8y3Svkw. [Zugriff am 22 February 2022].

[6] Boston Dynamics, „Youtube - Parcour Atlas," October 2018. [Online]. Available: https://www.youtube.com/watch?v=LikxFZZO2sk . [Zugriff am February 2022].

[7] Agility Robotics, „Youtube: Cassie: Dynamic Planning on Stairs," 20 February 2019. [Online]. Available: https://www.youtube.com/watch?v=qV-92Bq96Co. [Zugriff am 22 February 2020].

[8] K. Wilson und J. H. Korn, „Attention during lectures: Beyond ten minutes," Bd. 34, SAGE Publications Sage CA: Los Angeles, CA, 2007, pp. 85-89.

[9] M. Valdes-Sosa, J. Iglesias, R. Torres und N. Trujillo-Barreto, „Switching attention between the local and global levels in visual objects," Elsevier Oxford, 2014, pp. 165-177.

[10] C. Rosen, „The myth of multitasking," JSTOR, 2008, pp. 105-110.

[11] F. A. Arizal, M. A. Muhammad, G. P. Djausal und others, „Word Per Minute (WPM) Lampung Script Keyboard," *Tepian,* Bd. 1, Nr. 3, pp. 79-83, 2020.

[12] C. Coupé, Y. M. Oh, D. Dediu und F. Pellegrino, „Different languages, similar encoding efficiency: Comparable information rates across the human communicative niche," Bd. 5, American Association for the Advancement of Science, 2019, p. eaaw2594.

[13] A. Mehrabian, Nonverbal communication, Routledge, 2017.

[14] A. Goswami und P. Vadakkepat, Humanoid robotics: a reference, Springer, 2019.

[15] J. Tuszynski, „The dynamics of c-termini of microtubules in dendrites: A possible clue for the role of neural cytoskeleton in the functioning of the brain," Bd. 1, 2006.

[16] K. P. Cosgrove, C. M. Mazure und J. K. Staley, „Evolving knowledge of sex differences in brain structure, function, and chemistry," Bd. 62, Elsevier, 2007, pp. 847-855.

[17] P. Hartmann, A. Ramseier, F. Gudat, M. J. Mihatsch und W. Polasek, „Normal weight of the brain in adults in relation to age, sex, body height and weight," Bd. 15, 1994, pp. 165-170.

[18] J. G. Wolff, „How the SP System May Promote Sustainability in Energy Consumption in IT Systems," *Sustainability,* Bd. 13, Nr. 8, p. 4565, 2021.

[19] Oak Ridge Leadership Computing Facility, „Summit," [Online]. Available: https://www.olcf.ornl.gov/summit/. [Zugriff am 22 February 2022].

[20] Modha, Dharmendra S., „Introducing a brain-inspired computer," 2017. [Online]. Available: http://www.research.ibm.com/articles/brain-chip. [Zugriff am 22 February 2022].

[21] E. C. Hall, Journey to the moon: the history of the Apollo guidance computer, Aiaa, 1996.

[22] Better Scientific Software (BSSw), „Celebrating Apollo's 50th anniversary: When 100 flops/watt was a giant leap," [Online]. Available: https://bssw.io/blog_posts/celebrating-apollo-s-50th-anniversary-when-100-flops-watt-was-a-giant-leap. [Zugriff am 22 February 2022].

[23] PoliTo Culture, „Youtube: Costruire il Futuro | Ricerca e Innovazione," 10 November 2017. [Online]. Available: https://www.youtube.com/watch?v=rpjwhJmyacs&t=3679s. [Zugriff am 22 February 2022].

[24] Apple, „IPhone 11 - Technical Specifications," [Online]. Available: https://www.apple.com/iphone-11/specs/. [Zugriff am 22 February 2022].

[25] N. Bostrom und A. Sandberg, „Whole brain emulation: a roadmap,“ *Lanc Univ Accessed January,* p. 2015, 2008.

[26] Brownell, W. E., „How the ear works - nature's solutions for listening. The Volta review,“ *The Volta review,* Bd. 99, Nr. 5, p. 9, 1997.

[27] K. Nakadai, T. Lourens, H. G. Okuno und H. Kitano, „Active audition for humanoid,“ in *AAAI/IAAI,* 2000, pp. 832-839.

[28] L. Gustavsson, E. Marklund, E. Klintfors und F. Lacerda, „Directional Hearing in a Humanoid Robot,“ *Proceedings from Fonetik 2006 Lund, June 7–9, 2006,* p. 45, 2006.

[29] R. Viciana-Abad, R. Marfil, J. M. Perez-Lorenzo, J. P. Bandera, A. Romero-Garces und P. Reche-Lopez, „Audio-visual perception system for a humanoid robotic head,“ Bd. 14, Multidisciplinary Digital Publishing Institute, 2014, pp. 9522-9545.

[30] B. B. Monson, E. J. Hunter, A. J. Lotto und B. H. Story, „The perceptual significance of high-frequency energy in the human voice,“ *Frontiers in psychology,* Bd. 5, p. 587, 2014.

[31] M. V. Andrianopoulos, K. N. Darrow und J. Chen, „Multimodal standardization of voice among four multicultural populations: fundamental frequency and spectral characteristics,“ *Journal of Voice,* Bd. 15, Nr. 2, pp. 194-219, 2001.

[32] R. J. Baken und R. F. Orlikoff, Clinical measurement of speech and voice, Cengage Learning, 2000.

[33] Centers for Disease Control and Prevention, „What Noises Cause Hearing Loss?,“ 7 October 2019. [Online]. Available: https://www.cdc.gov/nceh/hearing_loss/what_noises_cause_hearing_loss.html. [Zugriff am 22 February 2022].

[34] IEEE Spectrum Robots, „Handle,“ 2021. [Online]. Available: https://robots.ieee.org/robots/handle/. [Zugriff am February 2022].

[35] Boston Dynamics, „Youtube: Introducing Handle,“ February 2017. [Online]. Available: https://youtu.be/-7xvqQeoA8c. [Zugriff am February 2022].

[36] Ascento, „ASCENTO,“ 2022. [Online]. Available: https://www.ascento.ch/. [Zugriff am February].

[37] ETH Zürich, „ASCENTO,“ 2022. [Online]. Available: https://www.ascento.ethz.ch/. [Zugriff am February 2022].

[38] Boston Dynamics Support Center, „Spot Specifications,“ 4 November 2021. [Online]. Available: https://support.bostondynamics.com/s/article/Robot-specifications. [Zugriff am 22 February 2022].

[39] IEEE Spectrum Robots, „Spot,“ September 2019. [Online]. Available: https://robots.ieee.org/robots/spotmini/. [Zugriff am 22 February 2022].

[40] M. Hutter, C. Gehring, D. Jud, A. Lauber, C. D. Bellicoso, V. Tsounis, J. Hwangbo, K. Bodie, P. Fankhauser, M. Bloesch und others, „Anymal-a highly mobile and dynamic quadrupedal robot,“ 2016, pp. 38-44.

[41] S. Kitano, S. Hirose, A. Horigome und G. Endo, „TITAN-XIII: sprawling-type quadruped robot with ability of fast and energy-efficient walking,“ Bd. 3, Springer, 2016, pp. 1-16.

[42] R. Playter, M. Buehler und M. Raibert, „BigDog,“ in *Unmanned Systems Technology VIII,* SPIE, 2006, pp. 896--901.

[43] MIT News | Massachusetts Institute of Technology, „MIT ‘Cheetah’ ROBOT Rivals Running Animals in Efficiency,“ 8 March 2013. [Online]. Available: https://news.mit.edu/2013/mit-cheetah-robot-0308. [Zugriff am 22 February 2022].

[44] M. Bjelonic, R. Grandia, O. Harley, C. Galliard, S. Zimmermann und M. Hutter, „Whole-body mpc and online gait sequence generation for wheeled-legged robots," in *2021 IEEE/RSJ International Conference on Intelligent Robots and Systems (IROS),* 2020.

[45] Swiss Mile, „Swiss Mile," January 2022. [Online]. Available: https://www.swiss-mile.com/. [Zugriff am February 2022].

[46] M. Bjelonic, C. D. Bellicoso, Y. de Viragh, D. Sako, F. D. Tresoldi, F. Jenelten und M. Hutter, „Keep rollin'—whole-body motion control and planning for wheeled quadrupedal robots}„" *IEEE Robotics and Automation Letters,* Bd. 4, Nr. 2, pp. 2116--2123, 2019.

[47] S. Seok, A. Wang, M. Y. Chuah, D. J. Hyun, J. Lee, D. M. Otten, J. H. Lang und S. Kim, „Design principles for energy-efficient legged locomotion and implementation on the MIT cheetah robot," *Ieee/asme transactions on mechatronics,* Bd. 3, Nr. 20, pp. 1117-1129, 2014.

[48] T. Miki, J. Lee, J. Hwangbo, L. Wellhausen, V. Koltun und M. Hutter, „Learning robust perceptive locomotion for quadrupedal robots in the wild," *Science Robotics,* Bd. 7, Nr. 62, p. eabk2822, 2022.

[49] M. Tranzatto, F. Mascarich, L. Bernreiter, C. Godinho, M. Camurri, S. Khattak, T. Dang, V. Reijgwart, J. Loeje, D. Wisth und others, „Cerberus: Autonomous legged and aerial robotic exploration in the tunnel and urban circuits of the darpa subterranean challenge," *arXiv preprint arXiv:2201.07067,* 2022.

[50] Smith Marshal and Marshal Smith Technology, „Atlas and SpotMini More Agile than EVER, Running and Climbing Stairs.," May 2018. [Online]. Available: https://www.industrytap.com/atlas-spotmini-agile-ever-running-climbing-stairs/45464. [Zugriff am February 2022].

[51] M. M. Samson, A. Crowe, P. L. De Vreede, J. A. G. Dessens, S. A. Duursma und H. J. J. Verhaar, „Differences in gait parameters at a preferred walking speed in healthy subjects due to age, height and body weight," Bd. 13, Springer, 2001, pp. 16-21.

[52] T. V. Barreira, D. A. Rowe, M. Kang und others, „Parameters of walking and jogging in healthy young adults," Bd. 3, 2010, p. 2.
